# Biochemical assessment of α-α-subunit interactions of Na_v_1.5 in a heterologous expression system

**DOI:** 10.1038/s41598-026-50463-9

**Published:** 2026-05-04

**Authors:** Oksana Iamshanova, Anne-Flore Hämmerli, Suberja Sundaralingam, Arbresh Seljmani, Sabrina Guichard, Maria Essers, Jean-Sébastien Rougier, Hugues Abriel

**Affiliations:** 1 Medi - Center for Medical Education, Bern, Switzerland; 2 Institute of Biochemistry and Molecular Medicine, University of Bern, Bühlstrasse 28, Bern, CH-3012 Switzerland

**Keywords:** Voltage-gated sodium channel, Na_v_1.5, Dimer, Oligomerization, Protein aggregation, Heterologous expression system, Biochemistry, Biophysics, Biotechnology, Cell biology, Molecular biology, Structural biology

## Abstract

In cardiomyocytes, Na_v_1.5, the predominant voltage-gated sodium channel α-subunit, is localized at specialized membrane microdomains within the intercalated disc and lateral membrane. Although Na_v_1.5 remodeling within these microdomains could cause pathological cardiac phenotypes, it remains unclear whether Na_v_1.5 channels could form direct interactions or rather exist as individual proteins in close proximity within the cluster. Importantly, heterologous overexpression of any protein, and especially of the large transmembrane channel Na_v_1.5, could be associated with the insufficiency of endoplasmic reticulum folding machinery, hence leading to aspecific protein aggregation indistinguishable from the genuine α-α-subunit interactions. In this study, we show that the interactions between heterologous Na_v_1.5 proteins depend on nascent N-linked glycosylation, are supported by non-native intermolecular disulfide bonds, and are likely predisposed to hydrophobic “stickiness”. In addition, we show strong interactions between the full-length Na_v_1.5 and its truncated peptides: N-terminal domain, all four transmembrane domains, as well as the intracellular linker between domains I and II. Taken together, we conclude that the heterologous expression system is not optimal for the identification of α-α-subunit interaction sites of Na_v_1.5, and this study needs further investigation in native tissues.

**Supplementary Information:**

The online version contains supplementary material available at 10.1038/s41598-026-50463-9.

## Introduction

Human voltage-gated sodium (Na^+^) channels (VGSCs) represent a family of proteins consisting of one pore-forming α-subunit (Na_v_1.x) and two distinct auxiliary β-subunits (Na_v_β)^1^ The pore-forming α-subunit is comprised of four transmembrane domains (I–IV), each of which has six transmembrane segments (S1–S6), where S1–S4 represent the voltage-sensor and S5 and S6 form the pore^2^. N- and C-terminals, as well as linkers between the transmembrane domain (hereafter named L1, L2, and L3) are intracellular^2^. The most distinguishable feature of VGSCs is their fast permeation of Na^+^ current (*I*_Na_) in response to membrane depolarizations of few millivolts (mV), followed by rapid inactivation. Therefore, VGSCs are first in line to respond to an initial depolarization and, hence, are the key regulators of cellular excitability. Within various tissues and cell types (e.g., neurons, neuromuscular junctions, and cardiomyocytes), VGSCs have been observed to form clusters^3^. The clustering of VGSCs is believed to enlarge their functional output, i.e., increase membrane responsiveness to electrical stimuli and conduction^3,4^. However, it remains unclear whether the clustering of VGSCs is supported by direct interactions between its subunits. Recent publications, using heterologous expression systems, suggest that the physical interaction between VGSCs may lead to an electrical coupling, which may be involved in the potentiation of the genesis of an electrical signal and in cardiac disorders such as the Brugada syndrome^5^. However, the functional study by Selimi et al., performed in both a heterologous expression system and native cells, challenges this model, raising questions about the relevance of the physical interaction observed in the heterologous expression system^6^. Furthermore, Tano et al., reported that VGSCs assemble into oligomers irrespective of their electrophysiological properties and functional coupling^7^.

While Na_v_β-subunits were shown to homo- and hetero-oligomerize^8^, α-subunits were thought to present as singular protein units clustered together, mainly due to specific membrane targeting^9^. Not long ago, this dogma was challenged by findings reporting direct α–α-subunit interactions. Specifically, human Na_v_1.1, Na_v_1.2, Na_v_1.5, and Na_v_1.7 were shown to homodimerize when heterologously expressed without Na_v_β-subunits^5,10–14^. Importantly, the bacterial α-subunit of VGSC was previously demonstrated to assemble as a dimer-of-dimers, consequently forming a functional ion-conducting homotetrameric channel^15^. Domain-swapping tetramerization of the bacterial α-subunit of VGSC was reportedly achieved by hydrophobic, polar, and charged amino acid residues mainly located within the S5 and S6 helices^16^. Higher-order oligomers were not observed for the prokaryotic α-subunit of VGSC until a recent study suggested inter-channel dimerization may occur through the voltage-sensing domain in the resting state^17^. In contrast, human Na_v_1.5 reportedly dimerizes through its intracellular L1 region between domains I and II^5^. L1 is a subject of multiple post-translational modifications, in particular, the α–α-subunit interaction region described for Na_v_1.5 (i.e., Arg493-Arg517) is comprised of at least one methylation site and several phosphorylation sites^18^. However, nothing is known yet about the dependence of Na_v_1.5 dimerization on its activation state or relationship to post-translational modifications.

Biosynthesis of Na_v_1.5 is a multistep fine-tuned process starting with *SCN5A* transcription, followed by the production of nascent peptide in the endoplasmic reticulum (ER), its maturation, and subsequent anchoring to the plasma membrane^19^. Typically, faulty and nonfunctional channels are trapped in the ER, triggering the unfolded protein response (UPR) that leads to the degradation of unwanted protein cargo^20^. UPR is especially important for adult cardiomyocytes, where native Na_v_1.5 is abundant, to maintain cellular functionality because their regenerative potential is significantly low^21^. Folding of Na_v_1.5 is regulated by the ER quality control machinery, comprised of chaperones and folding enzymes^19^. As the nascent polypeptide chain enters the ER, a core glycan is added to an Asn at a specific N-glycosylation site^20^. Monoglucosylated N-linked glycans are substrates for the lectin chaperones calnexin and calreticulin, which assist in oxidative folding^20^. Another family of ER-residing folding enzymes is the protein disulfide isomerase family, members of which catalyze disulfide formation, isomerization, or reduction between juxtaposed Cys^20^. Thus, rapid detection and elimination of unfolded nascent peptides in the ER are complicated due to their shared properties with folded unmature proteins, such as exposed hydrophobic patches or aggregation-prone regions^20^. Accordingly, accumulated misfolded proteins can aggregate into larger structures, leading to ER stress and UPR activation^20^. Given the complexity of membrane protein folding, one can hypothesize that in a heterologous expression system, where the host ER machinery may not be fully tailored for proper Na_v_1.5 processing, at least some portion of overexpressed channels may aggregate. Importantly, at this point, misfolded Na_v_1.5 aggregates are difficult to distinguish from true Na_v_1.5 oligomers because they also contain direct α-α-subunit interactions.

Taken together, the aim of our study was to critically assess α-α-subunit interactions of Na_v_1.5 in a heterologous expression system, striving for a better understanding of the VGSC dimerization phenomenon.

## Experimental procedures

### cDNA constructs

Template cDNA constructs and their derivatives are listed in Table S1.

### Cell culture and transfection

Cell culture was performed as previously published^14^. The human embryonic kidney cell line tsA201 (ECACC Cat# 96121229, RRID: CVCL_2737) was cultured up to 20 passages at 37 °C with 5% CO_2_ in Dulbecco’s Modified Eagle’s Medium (41965, Gibco™, Thermo Fisher Scientific, USA), supplemented with 2 mM L-glutamine (G7513, Sigma, USA), 50 U/mL penicillin-streptomycin (15140122, Gibco) and 10% heat-inactivated fetal bovine serum (10270-106, Lot 2440045, Gibco). When needed, cells were split using phosphate-buffered saline (PBS; 10010-015, Gibco) and 0.05% Trypsin-EDTA (25300-054, Gibco). Mycoplasma contamination status was tested weekly with a PCR Mycoplasma Test Kit I/C (PK-CA91-1096, Promokine, PromoCell GmbH, Germany). cDNA constructs (1–3 µg) were introduced into cells at 70–90% confluency by transfection with LipoD293™ (SL100668, SignaGen^®^ Laboratories, USA) at a ratio 1:3 (LipoD293™ µL : DNA µg). Cells were harvested 48 h after transfection unless stated otherwise.

### Protein extraction from cellular monolayers

The procedure was performed as previously published^14^. Pre-washed (PBS) adherent monolayers of cells were scraped in 2 mL of cold PBS (pH 7.4) and pelleted by centrifugation for 5 min at 200 × g at 4 °C. Cell pellets were lysed in lysis buffer (50 mM NaCl, 50 mM imidazole/HCl, 2 mM 6-aminohexanoic acid, 1 mM EDTA, pH 7) with the addition of cOmplete tablets EDTA-free (04693132001, Roche, Switzerland), 0.5 mM Na_3_VO_4_, 0.5 mM NaFl, 10 µg/mL aprotinin, 10 µg/mL leupeptin, 1 mM phenylmethylsulfonyl fluoride, and 1% digitonin (D141, Sigma) for 1 h at 4 °C. Lysates were centrifuged at 16,100 × g for 15 min at 4 °C. The obtained supernatant was taken for further analysis. Protein concentrations were determined by Coo Assay Protein Dosage Reagent (UPF86420, Uptima, Israel).

### Protein extraction from mouse heart

According to the Swiss Federal Animal Protection Law, all animal experiments were performed and approved by Bern’s Cantonal Veterinary Administration (license BE88 2022). This investigation conforms to the Guideline for the Care and Use of Laboratory Animals, published by the US National Institutes of Health (NIH publication no. 85 − 23, revised 1996). Mice were housed in a controlled, specific pathogen-free environment (23 ± 1 °C; humidity 60%; lights on 06:00 AM – 06:00 PM; food and water available ad libitum; enriched environment) with a maximum of 6 mice per cage.

This study was conducted and reported in accordance with the ARRIVE (Animal Research: Reporting of In Vivo Experiments) guidelines. A total of nine C57BL/6JRj mice (both female and male, aged 20–40 weeks) were used to assess protein expression. All procedures were performed in compliance with institutional and national ethical standards for animal experimentation.

Mice were deeply anesthetized with a single intraperitoneal injection of ketamine/xylazine (200/20 mg/kg body weight) combined with heparin (25,000 IE/5 ml; 4 ml/kg body weight) to prevent blood coagulation. As we overdose the anaesthetic, the procedure has to be done quickly after the loss of reflexes and after the first sign of respiratory depression (due to the overdose of Xylazine) to avoid any damage to the heart. Animals were sacrificed via thoracotomy (median sternotomy) to allow rapid access to the heart, which was immediately excised for subsequent experimental procedures.

For the isolation of cardiomyocytes, the excised heart was cannulated and mounted in a Langendorff system for retrograde perfusion at 37 °C. The heart was perfused free of blood (few minutes) with nominally Ca^2+^-free solution containing (mmol/L): 135 NaCl, 4 KCl, 1.2 MgCl_2_, 1.2 NaH_2_PO4, 10 HEPES, 11 glucose, pH 7.4 (NaOH). Next, the heart was perfused with the same solutions with 50 µM Ca^2+^ and 275U collagenase type II (275U/mg, CLS-2, Worthington, NJ, USA) till digestion. Following digestion, the ventricle was transferred to the aforementioned buffer with 100 µM Ca^2+^, minced into small pieces and triturate to liberate single ventricular myocytes by gentle pipetting, and filtered through a 100 μm nylon mesh. After supernatant was removed, cardiomyocytes were lysed in 1x lysis buffer, 1% digitonin for 1 h at 4 °C.

For the homogenization procedures, the excised heart was washed three times in five volumes of ice-cold PBS and put in one volume of 2x lysis buffer. For homogenization with Benchtop Bioprep-24 (Hangzhou Allsheng Instruments, China), 30 silica beads of 1.4 mm diameter (531607, Milian) and 10 silica beads of 2.8 mm diameter (531608, Milian) were added. The homogenization was performed in 6 cycles at 4260 rpm for 5 s (7 m/s linear speed) with an interruption of 30 s at 4 °C between the cycles. For homogenization with VD12 Polytron (VWR), the heart was subjected to 2 cycles of 5 s of homogenization on ice.

After homogenization, digitonin was added to make a final concentration of 1% and samples were left to lyse for 1 h at 4 °C. Lysates were centrifuged at 16,100 × g for 15 min at 4 °C. The obtained supernatant was taken for further analysis. Protein concentrations were determined by Coo Assay Protein Dosage Reagent (UPF86420, Uptima).

### Co-immunoprecipitation

The procedure was performed as previously published^14^. Anti-FLAG^®^ M2 Magnetic Beads (M8823, Sigma) and Pierce™ anti-HA Magnetic Beads (88836, Thermo Scientific) were used at a proportion of 1 µL of beads per 40 µg of total protein lysate. Protein lysates were diluted in Tris-buffered saline [TBS, 20 M Tris, 0.1 mM NaCl, pH 7.6 (HCl-adjusted)] and mixed with 0.05% TBS-Tween-20-prewashed antibody-coupled beads at 4 °C overnight. After thoroughly washing the beads with TBS, the co-immunoprecipitated protein complex was eluted with 4× LDS sample buffer (NP0007, Invitrogen, USA). Samples were then analyzed with an immunoblotting technique (see below).

### Cell surface biotinylation

The procedure was performed as previously published^14^. Adherent cell monolayers were gently washed three times with cold PBS (pH 8) and incubated, while slowly rocking, with 1 mg/mL EZlink™ Sulfo-NHS-SS-Biotin (21331, Thermo Fisher Scientific) in PBS (pH 8) for 45 min at 4 °C. Afterward, excess biotin was quenched by washing with 50 mM Tris-HCl (pH 8), followed by several washes with PBS (pH 7.4). Extracted protein lysates were mixed with Streptavidin Sepharose High-Performance Beads (GE Healthcare, USA) at 4 °C overnight. After thoroughly washing the beads with washing buffer [0.1% bovine serum albumin (BSA), 0.001% Tween-20, PBS, pH 7.4], the biotinylated fraction was eluted with 4× LDS sample buffer and analyzed with an immunoblotting technique (see below).

### Sodium dodecyl sulfate–polyacrylamide gel electrophoresis (SDS-PAGE) and immunoblotting analysis

The procedure was performed as previously published^14^. Protein lysates containing LDS sample buffer with or without 100 mM dithiothreitol (DTT) were loaded onto 4%–8% or 4%–12% Tris-acetate acrylamide gels and run at 60 V in Tris-acetate running buffer (50 mM Tris, 50 mM Tricine, 0.1% SDS, pH 8.3). Afterward, proteins were transferred to a nitrocellulose membrane using a Trans-Blot Turbo system (1704158, Bio-Rad, USA). After validation of successful protein transfer with Ponceau S solution (0.1% Ponceau S, 12.5% acetic acid), membranes were blocked with 5% BSA in TBS-T (TBS, 0.1% Tween-20) for 1 h at room temperature and then incubated with primary antibody dilutions at 4 °C overnight (Table 1). After incubating membranes with secondary antibodies for 1 h at room temperature, infrared fluorescent signals were revealed with a LI-COR Odyssey Infrared Imaging System (LI-COR Biosciences, USA) or FUSION FX7 Imaging System (Witec, Germany) and quantified with ImageJ software (Rasband, W.S., US National Institutes of Health, Bethesda, MD, USA).

Primary and secondary antibodies used in this study are listed in Table 1.

**Table 1:** Description of primary and secondary antibodies used in this study.

Antigen	Dilution	Host	Epitope	RRID	Source
BiP/GRP78	1:500	rabbit	residues surrounding Gly584 of human BiP	AB_2119845	3177, Cell Signaling Technology (USA)
FLAG	1:1000	mouse	DYKDDDDK	AB_262044	F1804, Sigma-Aldrich (USA)
GFP	1:1000	mouse	n/a	AB_390913	11,814,460,001, Roche (Switzerland)
GFP	1:1000	rabbit	Full-length denatured and non-denatured TurboGFP and CopGFP	n/a	AB514, Lot#51,402,250,467, Evrogen (Russia)
HA	1:500	mouse	YPYDVPDYA	n/a	ENZ-ABS118-0500, Enzo Life Sciences (USA)
HA	1:1000	rabbit	YPYDVPDYA	AB_2810986	ab137838, Abcam (UK)
Na_v_1.5	1:1000	rabbit	DTVSRSSLEMSPLAPV	n/a	generated by Pineda (Germany)
Na_v_β1	1:200	rabbit	CTGVQVAE	n/a	a gift from Isabelle Decosterd (Lausanne, Switzerland)
Na_v_β2	1:1000	rabbit	n/a	n/a	
Na_v_β3	1:200	rabbit	CSAVPVEE	n/a	
Na_v_β4	1:1000	rabbit	n/a	n/a	E90110A, Enogene, a gift from Isabelle Decosterd (Lausanne, Switzerland)
α1-Na^+^/K^+^ ATPase	1:500	mouse	Full length native α1-Na^+^/K^+^ ATPase	AB_306023	ab7671, Abcam (UK)
α1-syntrophin	1:1000	rabbit	SGRRAPRTGLLELRAC	n/a	generated by Pineda (Germany)
α-actin	1:1000	rabbit	SGPSIVHRKCF	AB_476693	A2066, Sigma-Aldrich
calnexin	1:1000	rabbit	C-terminus of human calnexin (amino acids 573–592)	AB_476845	C4731, Sigma-Aldrich
IgG mouse	1:20,000	goat	Reacts with the heavy and light chains of mouse IgG_1_, IgG_2a_, IgG_2b_, and IgG_3_, and with the light chains of mouse IgM and IgA.	AB_10956588	926–68070, LI-COR Biosciences (Germany)
IgG rabbit	1:20,000	goat	Reacts with the heavy and light chains of rabbit IgG, and with the light chains of rabbit IgM and IgA.	AB_621843	926–32211, LI-COR Biosciences

### Protein-protein interaction assay in living cells

The NanoLuc^®^ Binary Technology NanoBiT^®^ (Promega, USA) allowed us to monitor protein-protein interactions between two wild-type (WT) Na_v_1.5 α-subunits in living cells as described previously^14^. Cells were plated at 30 × 10^3^ cells/well into white bottom opaque 96-well plates (3917, Corning), left for at least 4 h in the incubator at 37 °C with 5% CO_2_ to settle down, and transfected with NanoBiT constructs (Table 1). 48 h after transfection, the luminescent signal was measured with a Nano-Glo^®^ Live Cell Assay (N2012, Promega) and normalized to the cell quantity represented by the fluorescent signal obtained with a CellTiter-Fluor^®^ Cell Viability Assay (G6082, Promega). Signals were detected with a GloMax^®^ Explorer Multimode Microplate Reader (GM3500, Promega). Importantly, in the absence of specific interacting partners LgBiT and SmBiT have low affinity for self-association (K_D_ = 190 µM).

### Data and statistical analysis

Data are represented as the mean ± SEM or SD. Data normality was tested using the Shapiro-Wilk test. Since all datasets were normally distributed, comparisons were performed with ordinary one-way ANOVA and Tukey’s multiple comparisons tests. In cases where data were normalized to a control condition, the statistical significance was calculated with a one-sample two-tailed t-test with hypothetical mean value = 1 (Prism version 8.4.3; GraphPad, CA, USA). Exact *p*-values are indicated in the figures. Neither randomization nor blinding were performed in this study.

## Results

### Heterologous expression of Na_V_1.5 introduces α-α-subunit interactions, whereas native tissue extracts do not reveal such interactions

Previous studies reported the α-α-subunit interactions of Na_v_1.5^5,10,14^. It is important to note that the “true” Na_v_1.5 dimers/oligomers and the aggregates of Na_v_1.5 would both possess α-α-subunit interactions. For clarification, protein oligomers are discrete complexes with specific structures, assembling as two protein subunits, called dimers, three – trimers, four – tetramers and etc. In contrast, protein aggregates are disordered protein clusters resulting from misfolding and accumulation. Hereafter, we use the definition of oligomer alongside protein aggregate, as those two are indistinguishable by the biochemical methods used in this study.

**Fig. 1:**
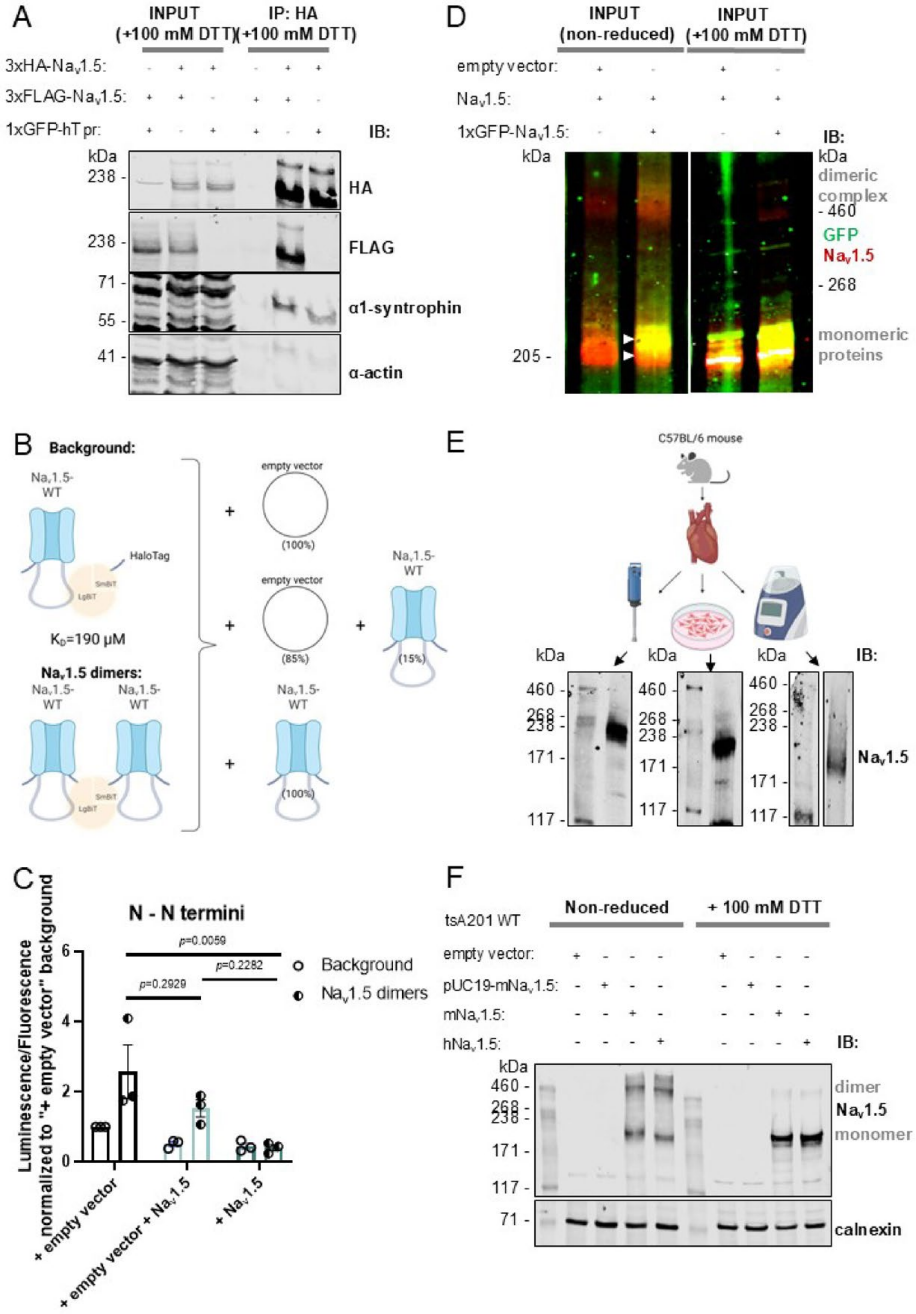
In a heterologous expression system, Na_v_1.5 proteins co-immunoprecipitate and interact in living cells. Na_v_1.5 dimers are expressed at the cell surface and in the cytoplasm, can be visualized in non-reducing SDS-PAGE, and are disrupted by DTT. **(A)** Immunoblot of reduced total lysate (“INPUT”) and HA-specific immunoprecipitated (“IP: HA”) fractions from tsA201 cells 48 h after transient co-expression of 3xHA-Na_v_1.5, 3xFLAG-Na_v_1.5, and 1xGFP-hTpr. Nuclear basket protein, which had a similarly sized plasmid, was used as a transfection control. Immunoprecipitation was performed with HA-coupled magnetic beads. Endogenous α1-syntrophin was used as a positive control for co-immunoprecipitation with Na_v_1.5, and α-actin was a negative control. **(B)** Schematic illustration of the NanoBiT assay, whereby different quantities of untagged Na_v_1.5 are co-expressed together with the luminesce-producing pair of Na_v_1.5-LgBiT and Na_v_1.5-SmBiT. Background signal was determined by co-expression of Na_v_1.5-LgBiT with a non-interacting control, HaloTag-SmBiT. **(C)** In living cells, the presence of untagged Na_v_1.5 significantly decreased the luminescence produced by the interaction between Na_v_1.5-LgBiT and Na_v_1.5-SmBiT. This signifies that untagged Na_v_1.5 competed for the interaction between Na_v_1.5-LgBiT and Na_v_1.5-SmBiT, therefore the observed luminescence signal was Na_v_1.5-specific. Results of the NanoBiT assay are presented as the relative intensity of luminescence (indicating the level of protein-protein interactions) normalized to the fluorescence (indicating the number of cells) of tsA201 cells 48 h after transfection. Each dataset was normalized to “+ empty vector” control. Data are presented as mean ± SEM from three biological replicates. Multiplicity-adjusted *p*-values, calculated with one-way ANOVA and post hoc Tukey’s multiple comparisons, are indicated in each panel. **(D)** Immunoblot of the non-reduced and reduced total lysate from tsA201 cells 48 h after transient expression of untagged Na_v_1.5 alone and with 1xGFP-Na_v_1.5. White triangles indicate bands corresponding to monomeric untagged Na_v_1.5 and monomeric 1xGFP-Na_v_1.5. **(E)** Immunoblots of native Na_v_1.5 obtained from non-reduced lysates of excised mouse hearts prepared using a benchtop homogenizer (left panel), direct lysis of isolated cardiomyocytes (middle panel), and a tissue homogenizer (right panel). **(F)** Immunoblot of non-reduced and reduced total lysates of tsA201 cells 48 h after transient co-expression of mouse and human Na_v_1.5.

Here, we confirmed that differently tagged Na_v_1.5 proteins co-immunoprecipitated in a heterologous expression system represented by tsA201 cells (Fig. 1A). α-syntrophin is a known protein partner of Na_v_1.5 and is endogenously expressed in tsA201 cells^24,25^. Therefore, α-syntrophin was used in this experiment as a positive control to confirm successful immunoprecipitation of Na_v_1.5. The reducing agent, DTT, was added at standard concentration 100 mM to the final eluate after immunoprecipitation, as well as to the input. In living cells, the dimerization of Na_v_1.5 was preserved^14^. Because interactions through the N-N termini of Na_v_1.5 consistently produced the brightest signal^14^, they were used as a default configuration for the NanoBiT assay in this study. To read-out background luminescence Na_v_1.5-LgBiT was co-expressed with HaloTag-SmBiT, which were expected to interact with low affinity (K_D_=190 µM). The untagged Na_v_1.5 (at different ratios to empty vector, pcDNA3.1-CMV, as 100%, 15%, and 0%) was co-transfected with background pair (Na_v_1.5-LgBiT + HaloTag-SmBiT) and pair expected to produce Na_v_1.5 dimers (Na_v_1.5-LgBiT + Na_v_1.5-SmBiT) (Fig. 1B). Based on results showing that untagged Na_v_1.5 competed for its interaction with the luminescence-producing pair of Na_v_1.5-LgBiT and Na_v_1.5-SmBiT (Fig. 1B, C) but not the control luminescence-producing pair of well-known interacting proteins PRKAR2A-LgBiT and PRKACA-SmBiT (Fig. S1), we concluded that the observed dimerization was specific to Na_v_1.5. To resolve Na_v_1.5 dimers at the expected molecular weight of ~ 460 kDa, we performed non-reducing 4%–8% gradient SDS-PAGE. To deduce whether the observed high molecular weight band represented the Na_v_1.5-Na_v_1.5 complex rather than an aspecific band recognized by the antibody used during immunoblotting, we co-expressed non-tagged Na_v_1.5 with green fluorescent protein (GFP)-tagged Na_v_1.5. Accordingly, a ~ 460-kDa band was revealed with both antibodies, against Na_v_1.5 and against GFP, while differences in the sizes of corresponding monomeric Na_v_1.5 proteins (~ 30 kDa for GFP tag) confirmed the validity of the constructs used (Fig. 1D). Therefore, we concluded that the ~ 460-kDa band represented the dimer of Na_v_1.5 proteins, which could also be represented by the SDS-resistant aggregate.

Because our non-reducing SDS-PAGE yielded robust results for detecting the putative Na_v_1.5 dimer/aggregated, we decided to apply this method to native tissues. Using a C57BL/6 mouse model with two untagged wild-type alleles for *SCN5A*, we performed three different types of protein extraction on excised hearts: tissue homogenizer, benchtop homogenizer, and direct lysis of isolated cardiomyocytes. Importantly, regardless of the protein extraction method used, only monomers of Na_v_1.5 were detected (Fig. 1E). To verify that our result was not caused by species specificity, we carried out non-reducing SDS-PAGE on protein lysates from tsA201 cells in parallel expressing mouse and human Na_v_1.5 proteins (Fig. 1F). Immunoblotting analysis revealed the Na_v_1.5-specific bands corresponding to the expected size of its monomeric state (between 171-kDa and 238-kDa of protein standard bands) as well as its putative dimeric/aggregate state (~ 460-kDa of protein standard band) observed in Fig. 1D (Fig. 1F). As expected, the cloning vector pUC19 did not express *Scn5a*, and hence the monomeric and putative dimeric bands expressed by pcDNA3.1-hSCN5A-WT and pcDNA3.1-mScn5a-WT were specific to Na_v_1.5 (Fig. 1F). Interestingly, application of a reducing agent, 100 mM DTT, decreased the intensity of putative Na_v_1.5 dimer/aggregate band and increased the amount of Na_v_1.5 monomers (Fig. 1F). This could be due to the disruption of Na_v_1.5 dimers into monomers, suggesting the involvement of covalent disulfide bridges. Therefore, we concluded that these putative Na_v_1.5 dimers/aggregates were at least partially sensitive to the reducing agent and were absent or not detectable under our experimental conditions in non-diseased mouse cardiomyocytes.

### Na_v_1.5 dimers are present at the plasma membrane, but are not primarily formed by disulfide bonds

**Fig. 2:**
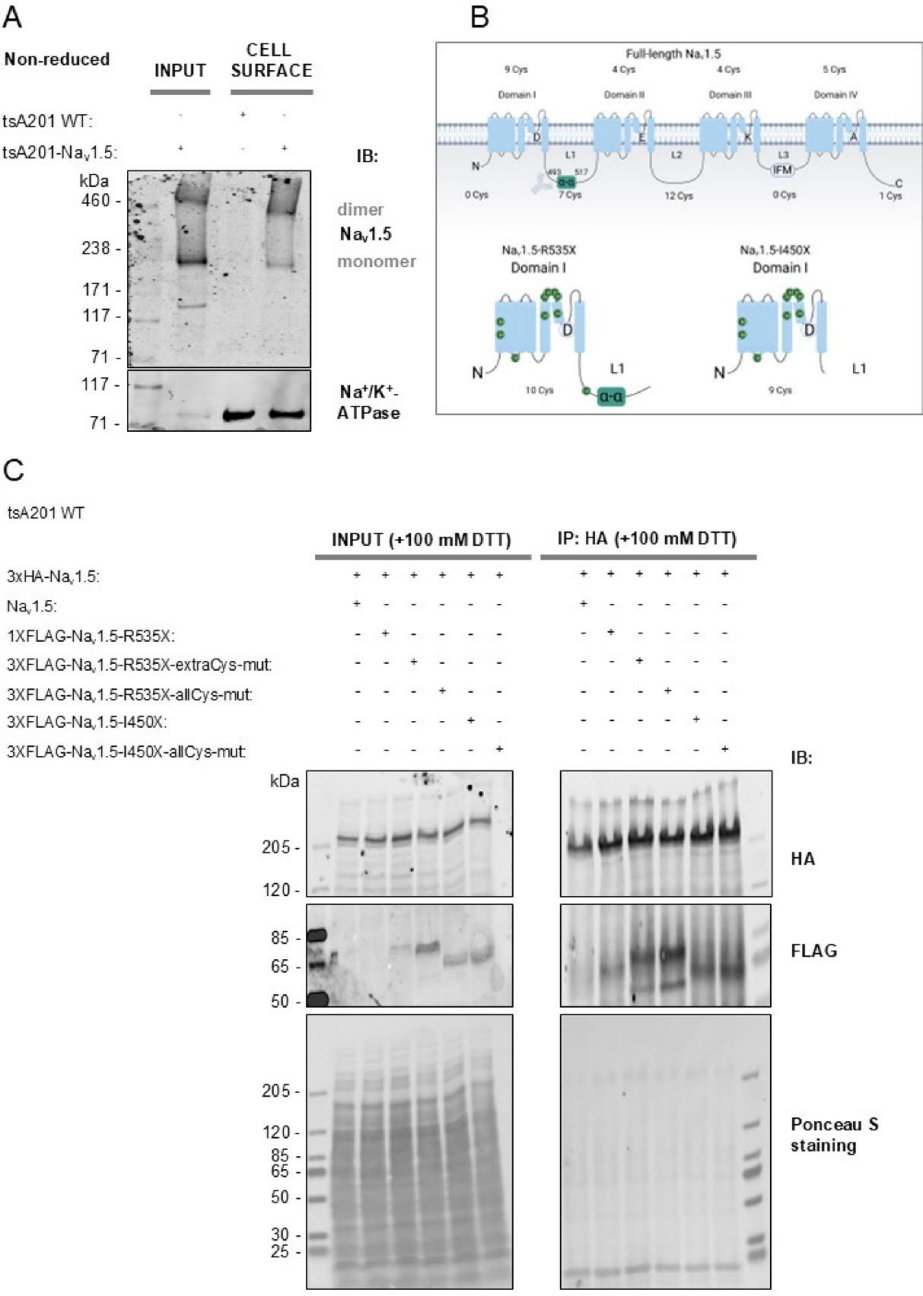
Disulfide bridges are involved in oligomerization of Na_v_1.5 proteins but are not exclusive to this process. **(A)** Immunoblot of non-reduced total lysate (“INPUT”) and biotinylated (“CELL SURFACE”) fractions of wild-type (WT) and tsA201 cells stably expressing Na_v_1.5. Dimers and monomers of Na_v_1.5 were revealed with an anti-Nav1.5 antibody. Endogenous α1-Na^+^/K^+^-ATPase was used as a positive control for the biotinylated fraction. **(B)** Schematic illustration of full-length Na_v_1.5 with numbers of Cys corresponding to each domain indicated, and truncated Na_v_1.5 constructs with total numbers of Cys. Na_v_1.5-R535X comprises the presumable α-α-subunit interaction site between Arg493-Arg517, while Na_v_1.5-I450X does not include this site. **(C)** Immunoblot of the reduced total lysate (“INPUT”) and HA-specific immunoprecipitated (“IP: HA”) fractions of tsA201 cells 48 h after transient co-expression of 3xHA-Na_v_1.5, untagged Na_v_1.5, 1xFLAG-Na_v_1.5-R535X, 3xFLAG-Na_v_1.5-R535X-extraCys-mut, 3xFLAG-Na_v_1.5-R535X-allCys-mut, 3xFLAG-Na_v_1.5-I450X, and 3xFLAG-Na_v_1.5-I450X-allCys-mut. Ponceaus S staining was used as a loading control.

Functional Na_v_1.5 proteins are typically targeted to the plasma membrane. To assess whether the interacting α-subunits were trapped intracellularly, we performed a biotinylation assay. Our results suggest that both monomers and putative dimers/aggregates of Na_v_1.5 were present at the cell surface when heterologously expressed in tsA201 (Fig. 2A).

Since Cys bridges are typically extracellular, this finding was in line with our previous suggestion that disulfide bridges may play a role in linking two Na_v_1.5 proteins together. However, considering that α-α-subunit interactions could also be represented by the aggregated misfolded proteins, we also assessed the possibility of non-native disulfide bridges formed via intracellular Cys.

The full-length Na_v_1.5 (UniProtKB: Q14524-1) has 42 Cys residues in total (Fig. 2B). Importantly, a previous study reported on the existence of a direct α-α-subunit interaction site between Arg493-Arg517 (Fig. 2B)^5^. Like that study, we used the truncated Na_v_1.5 proteins, Na_v_1.5-Arg535X and Na_v_1.5-Ile450X, to additionally investigate the role of Arg493-Arg517 for the direct interaction (Fig. 2B). Moreover, to identify which Cys could be responsible for the intermolecular disulfide bonds between Na_v_1.5 proteins, we mutated Cys into Ala. For this, we created Na_v_1.5-Arg535X constructs with only 4 extracellular Cys (Na_v_1.5-Arg535X-extraCys-mut) and all 10 Cys being mutated (Na_v_1.5-Arg535X-allCys-mut). Similarly, we mutated all 9 Cys of Na_v_1.5-Ile450X (Na_v_1.5-Ile450X-allCys-mut). Co-immunoprecipitation analysis between full-length Na_v_1.5 and the aforementioned truncated Na_v_1.5 proteins revealed that mutating only 4 extracellular Cys or all 10 Cys was insufficient to abolish the ability of Na_v_1.5-Arg535X protein to interact with the full-length Na_v_1.5 (Fig. 2C). Furthermore, Na_v_1.5-Ile450X, lacking the presumed α-α-subunit interaction site between Arg493-Arg517, also preserved its binding with the full-length Na_v_1.5, and this binding was not affected by the absence of Cys (Fig. 2C). Therefore, we concluded that Arg493-Arg517 was not responsible for linking two Na_v_1.5 proteins. Moreover, although Na_v_1.5 multimers/aggregates could have been at least partially supported via disulfide bridges, as observed in Fig. 1F, they were not the main cause of its oligomerization/aggregation.

### Na_v_1.5 dimers are unaffected by co-expression with Na_v_β-subunits

**Fig. 3:**
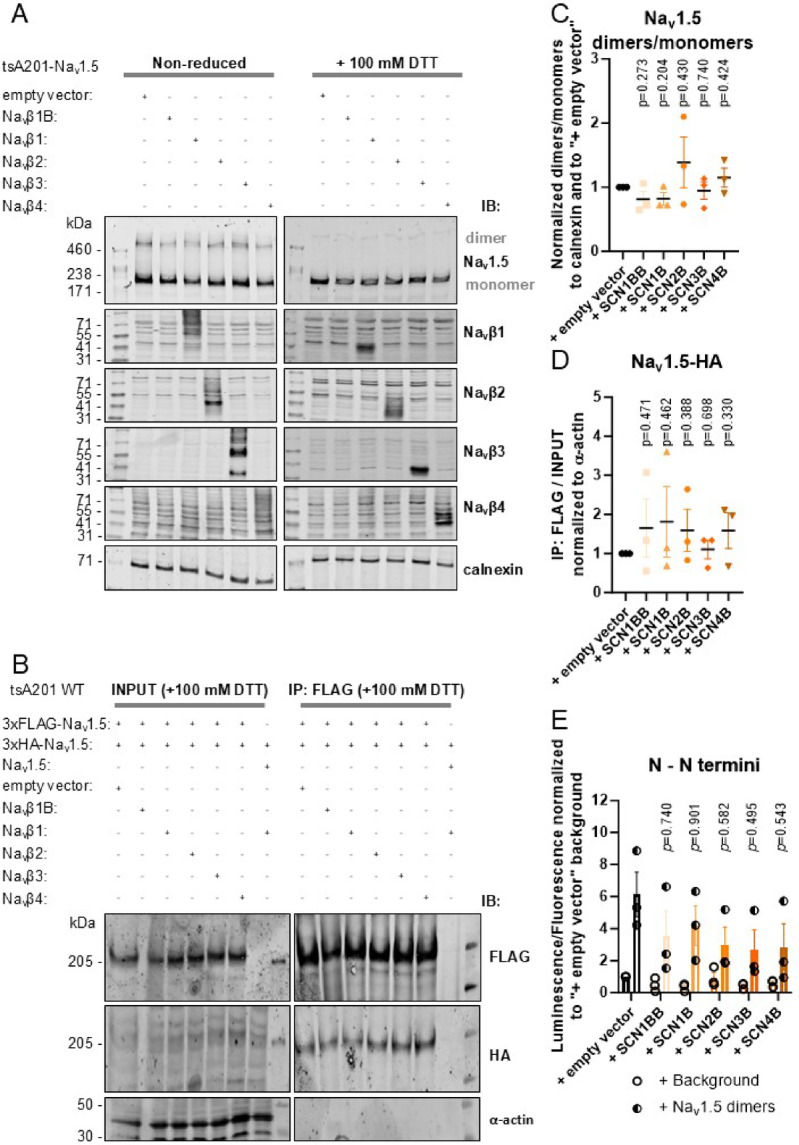
Na_v_1.5 dimers are unaffected by co-expression of Na_v_β-subunits. **(A)** Immunoblot of non-reduced and reduced total lysate fractions of tsA201 cells stably expressing Na_v_1.5 after 48 h of transient expression of Na_v_β1B, Na_v_β1, Na_v_β2, Na_v_β3, and Na_v_β4. Dimers and monomers of Na_v_1.5 were revealed with anti-Na_v_1.5. Endogenous calnexin was used as a loading control. Of note, Na_v_β1B is not expected to have an epitope for detection with anti-Na_v_β1. **(B)** Immunoblot of the reduced total lysate (“INPUT”) and FLAG-specific immunoprecipitated (“IP: FLAG”) fractions of tsA201 cells 48 h after transient co-expression of 3xHA-Na_v_1.5 and 3xFLAG-Na_v_1.5 with and without Navβ1B, Navβ1, Navβ2, Navβ3, and Navβ4. Endogenous α-actin was used as a negative control. **(C)** Intensities of Na_v_1.5 dimers/monomers from (A) were normalized to calnexin and the control condition (“+ empty vector”). Data are presented as mean ± SEM from three biological replicates. Individual *p*-values, calculated with a one-sample two-tailed t-test with hypothetical mean value = 1, are indicated in each panel. **(D)** Intensities of Na_v_1.5-HA in (B) from “IP: FLAG” were normalized to their corresponding intensities from INPUT and further normalized to calnexin and the control condition (“+ empty vector”). Data are presented as mean ± SEM from three biological replicates. Individual *p*-values, calculated with a one-sample two-tailed t-test with hypothetical mean value = 1, are indicated in each panel. **(E)** Results of the NanoBiT assay are presented as the relative intensity of luminescence (indicating the level of protein-protein interactions) normalized to fluorescence (indicating the number of cells) of tsA201 cells 48 h after transfection. Each dataset was normalized to “+ empty vector” control. Data are presented as mean ± SEM from three biological replicates. The multiplicity-adjusted *p*-values, calculated with one-way ANOVA and post hoc Tukey’s multiple comparisons, are indicated in each panel.

Na_v_β-subunits, particularly Na_v_β2 and Na_v_β4, are known to form intermolecular disulfide bridges within themselves as well as with α-subunits of VGSCs [reviewed in^26^. Notably, a previous study proposed that α-α-subunit interactions of Na_v_1.5 were regulated by the presence of Na_v_β1^12^. Although we and other groups reported the self-interactions of Na_v_1.5 proteins in the absence of Na_v_β-subunits^5,14^, we decided to revise this question in this study. First, we compared the ratios of Na_v_1.5 dimers/monomers with and without individual co-expression of Na_v_β1B, Na_v_β1, Na_v_β2, Na_v_β3, and Na_v_β4 subunits (Fig. 3A, C). Of note, in reduced condition Na_v_β subunits appeared at their expected glycosylated sizes of 35–40 kDa, while in non-reduced condition they exhibited higher molecular weight of 60–80 kDa, confirming the existence of homo-intramolecular disulfide bonds within their extracellular Ig-like domains (Fig. 3A). Second, we analyzed the amount of haemagglutinin (HA)-tagged Na_v_1.5 that co-immunoprecipitated with FLAG-Na_v_1.5 in the absence and presence of Na_v_β-subunits co-expression (Fig. 3B, D). Third, we estimated levels of the putative Na_v_1.5 dimers between the aforementioned conditions in living cells (Fig. 3E). Because we did not observe an effect in any experimental procedure, we concluded that Na_v_β-subunits are indeed dispensable for the homo-oligomerization/aggregation of Na_v_1.5 in our heterologous expression system.

### Transmembrane domains strongly interact with full-length Na_v_1.5 and reduce its protein level

**Fig. 4:**
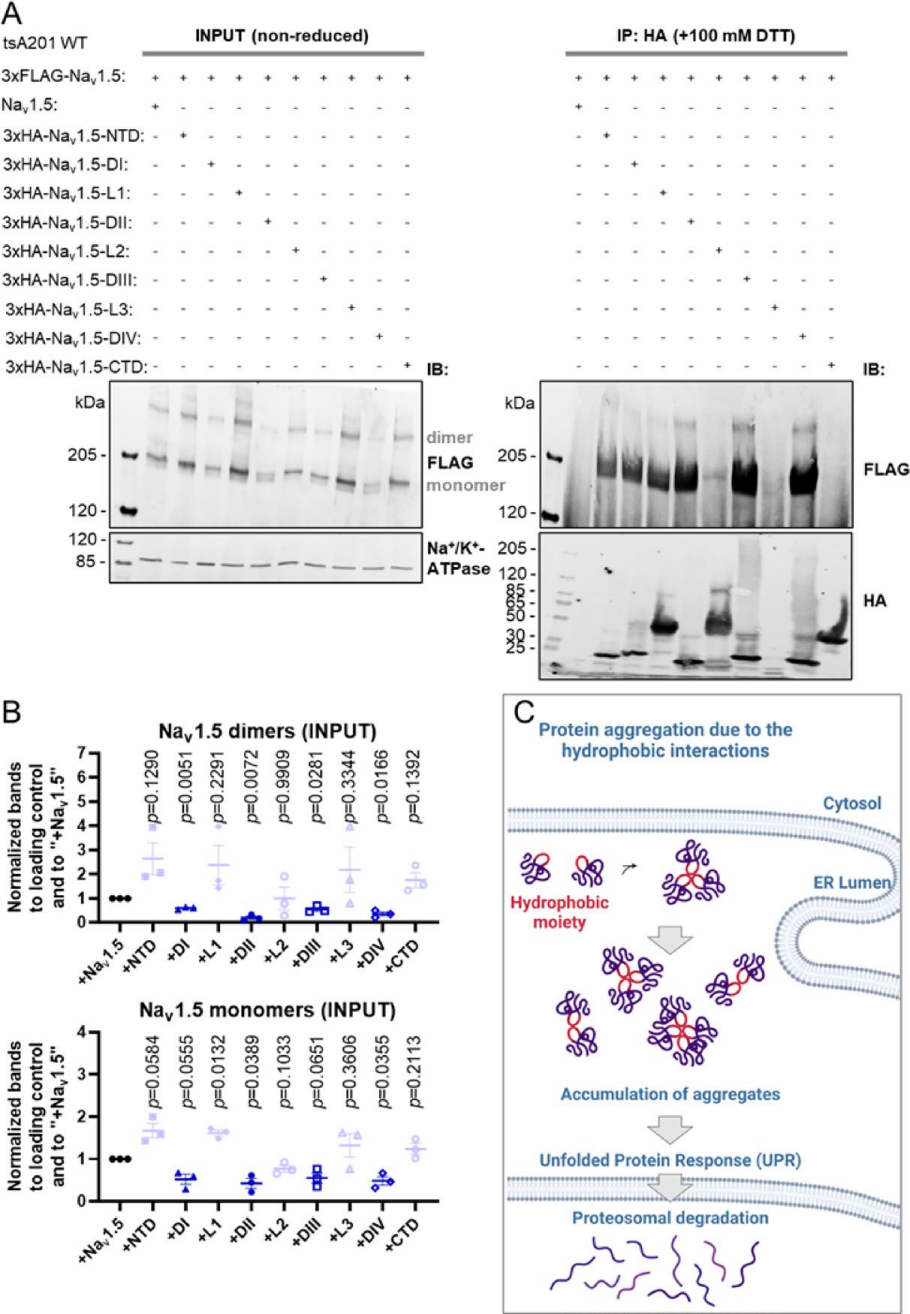
α-α-Subunit interactions occur between full-length Na_v_1.5 and all four transmembrane domains, the N-terminal domain, and the linker between domains I and II. **(A)** Immunoblot of non-reduced total lysate (“INPUT”) and reduced HA-specific immunoprecipitated (“IP: HA”) fractions of tsA201 cells 48 h after transient co-expression of 3xFLAG-Na_v_1.5 with untagged Na_v_1.5, 3xHA-Na_v_1.5-NTD, 3xHA-Na_v_1.5-DI, 3xHA-Na_v_1.5-L1, 3xHA-Na_v_1.5-DII, 3xHA-Na_v_1.5-L2, 3xHA-Na_v_1.5-DIII, 3xHA-Na_v_1.5-L3, 3xHA-Na_v_1.5-DIV, and 3xHA-Na_v_1.5-CTD. In total lysate, Na_v_1.5 dimers and monomers were revealed with an anti-FLAG antibody. The immunoprecipitated domains of Na_v_1.5 were revealed with an anti-HA antibody, while co-immunoprecipitated full-length Na_v_1.5 proteins were revealed with an anti-FLAG antibody. Endogenous α1-Na^+^/K^+^-ATPase was used as a loading control for the total lysate fraction. **(B)** Intensities of non-reduced Na_v_1.5 dimers and non-reduced Na_v_1.5 monomers from (A) were normalized to α1-Na^+^/K^+^-ATPase and the condition with co-expression of untagged full-length Na_v_1.5 (“+Na_v_1.5”). Data are presented as mean ± SEM from three biological replicates. Individual *p*-values, calculated with a one-sample two-tailed t-test with hypothetical mean value = 1, are indicated in each panel. **(C)** Schematic illustration of how hydrophobic interactions between transmembrane domains of Na_v_1.5 might promote protein aggregation and UPR.

Because our data did not confirm the exclusivity of the previously described Arg493-Arg517 site for α-α-subunit interactions of Na_v_1.5 (Fig. 2C)^5^, we decided to revise the interaction sites by co-immunoprecipitation analysis between different parts of Na_v_1.5 and the full-length protein (Fig. 4A). Strikingly, the presence of transmembrane domains I–IV decreased total levels (both putative dimers/aggregates and monomers) of full-length Na_v_1.5 (Fig. 4A, B). This could be explained by increased aspecific hydrophobic interactions between the large transmembrane domains of Na_v_1.5 that would lead to protein aggregation and activation of UPR with subsequent proteasomal degradation (Fig. 4C). Indeed, although the total protein quantity was diminished, the interaction between transmembrane domains I–IV and full-length Na_v_1.5 remained very strong (Fig. 4B). Furthermore, we detected potent interactions between full-length Na_v_1.5, its intracellular N-terminal domain (NTD), and the linker between domains I and II (named L1) (Fig. 4B). Overall, these results indicate that the heterologously expressed Na_v_1.5 proteins interact with each other not due to a specific amino acid sequence but rather within multiple regions.

### Na_v_1.5 dimerization levels are regulated by its protein expression level and core N-linked glycosylation in the ER

**Fig. 5:**
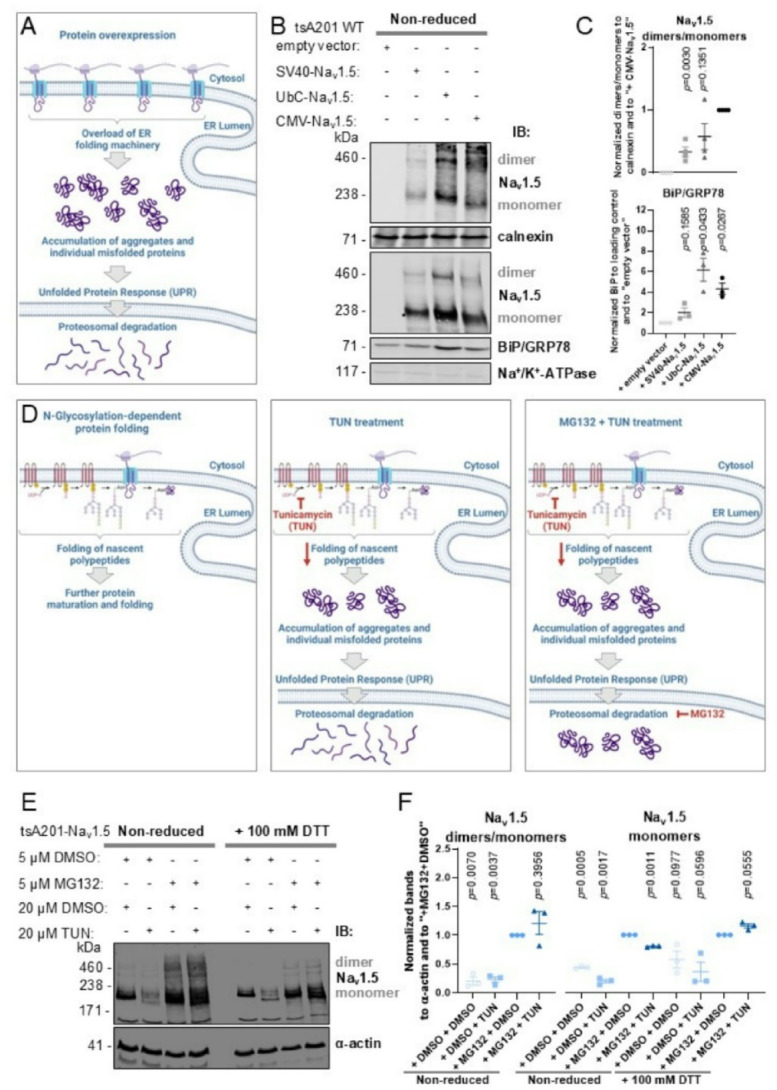
Na_v_1.5 dimerization is increased at higher expression levels and with ER stress induction. **(A)** Schematic illustration of how protein overexpression might overload ER folding machinery and trigger UPR. **(B)** Immunoblot of the non-reduced total lysate fraction of tsA201 cells after 48 h of transient expression of Na_v_1.5 with promoters of different strengths. Dimers and monomers of Na_v_1.5 were revealed with anti-Na_v_1.5. Endogenous calnexin was used as a loading control. **(C)** Intensities of Na_v_1.5 dimers/monomers from (B) were normalized to calnexin and the condition with the strongest promoter (“+CMV-Na_v_1.5”). Data are presented as mean ± SEM from three biological replicates. Individual *p*-values, calculated with a one-sample two-tailed t-test with hypothetical mean value = 1, are indicated in each panel. **(D)** Schematic illustration of how TUN treatment (prevents N-core glycosylation) and MG132 treatment (inhibits proteasomal degradation) affect protein folding and UPR. **(E)** Immunoblot of non-reduced and reduced total lysate fractions of tsA201 cells stably expressing Na_v_1.5 with the indicated combinations of TUN and MG132 treatments. Endogenous α-actin was used as a loading control. **(F)** Intensities of non-reduced Na_v_1.5 dimers/monomers, as well as non-reduced and reduced Na_v_1.5 monomers from (B), were normalized to α-actin and the condition with inhibited proteasomal degradation (“+MG132 + DMSO”). Data are presented as mean ± SEM from three biological replicates. Individual *p*-values, calculated with a one-sample two-tailed t-test with hypothetical mean value = 1, are indicated in each panel.

Mercier et al., showed that in HEK293T cells co-immunoprecipitation between Na_v_1.5 WT and Arg1432Gly mutant was dependent on co-expression with Na_v_β1^12^. Another group showed that Na_v_1.5 WT co-immunoprecipitated with WT as well as with Arg104Trp and Gly1642X mutants while being expressed in HEK293 cells^10,13^. In their further study, Na_v_1.1, Na_v_1.2, and Na_v_1.5 dimers were resolved by SDS-PAGE after crosslinking in HEK293 cells^5^. Furthermore, WT and Leu325Arg mutant of Na_v_1.5 were shown to co-immunoprecipitate, to be able to cross-link and to form SDS-sensitive dimers in Blue Native PAGE in HEK293 cells^27^. Moreover, interactions between Na_v_1.5 proteins were demonstrated by TIRF/FRET imaging^5^. Rühlmann et al., reported that Na_v_1.5 and Na_v_1.7 expressed in *Xenopus* oocytes migrated as non-covalent homodimers under native PAGE^11^. Tano et al., showed co-immunoprecipitation between Nav1.5 WT, Gly833Arg, and Thr1396Pro mutants in HEK293^7^. Nevertheless, all α-α-subunit interactions of VGSCs were previously shown in a heterologous overexpression system^5,7,10–14,27^. In this case, there is a possibility that the overload and/or unsuitability of protein folding machinery could lead to the aggregation of ectopic proteins (Fig. 5A). Thus, we decided to assess the oligomerization/aggregation of Na_v_1.5 while controlling the level of its overexpression. To achieve this, we compared the ratios of the putative reducing agent-sensitive Na_v_1.5 dimers/aggregates to its monomers produced under promoters with different expression strengths (Fig. 5B). According to our results, the Na_v_1.5 dimer/monomer ratio was directly proportional to the level of heterologous protein expression (Fig. 5C). Furthermore, ER stress marker, BiP/GRP78 was significantly upregulated in condition of moderate and strong promoters, Ubiquitin C (UbC) and cytomegalovirus (CMV), but not weak promoter, SV40, when compared to transfection with an empty vector (Fig. 5B, C). This suggests that higher Na_v_1.5 expression is associated with increased ER stress. Interestingly, the strongest promoter, CMV, also led to the production of monomeric Na_v_1.5 of a smaller size compared with monomeric Na_v_1.5 proteins produced with SV40 and UbC promoters (Fig. 5B). This encouraged us to further investigate whether a slight change in the size of monomeric Na_v_1.5 under strong levels of expression was associated with the insufficiency of post-translational modifications resulting from saturated protein folding machinery (Fig. 5A). One of the first post-translational modifications is N-glycosylation, which occurs while the nascent peptide is being synthesized to assist with correct protein folding in the ER (Fig. 5D). Both native and heterologous Na_v_1.5 were shown to be N-glycosylated^28^. Therefore, we used an inhibitor of N-core glycosylation, tunicamycin (TUN), to test its importance for the heterologous oligomerization/aggregation of Na_v_1.5 (Fig. 5D). TUN prevents the addition of oligosaccharides to nascent polypeptides, leading to their misfolding and accumulation in the ER (Fig. 5D). This, in turn, induces ER stress followed by activation of the UPR and proteasomal degradation (Fig. 5D). To constrain the latter and preserve Na_v_1.5 dimers/aggregates possibly evoked due to the affected N-glycosylation, we used the cell-permeable proteasomal inhibitor MG132 (Fig. 5D). Because the presence of TUN and MG132 might significantly alter the total amount of Na_v_1.5 protein, we compared the differences between Na_v_1.5 dimer/monomer ratios under various conditions by normalizing them to treatment with the highest total protein amount, i.e. MG132 alone. Accordingly, our results confirmed that TUN alone induced the degradation of heterologous protein and reduced the size of the band corresponding to monomeric Na_v_1.5, whereas the addition of MG132 rescued the amount of heterologous Na_v_1.5 (Fig. 5E, F). Furthermore, the amount of non-reduced Na_v_1.5 monomers decreased under the condition of combined TUN and MG132 treatment, while the reduced Na_v_1.5 monomers tended to increase (Fig. 5E, F). This result could signify that the combination of TUN with MG132 facilitated the accumulation of Na_v_1.5 oligomers compared with MG132 treatment alone. Altogether, our results suggest that a high production rate of heterologous Na_v_1.5 might saturate the ER folding machinery, and this, in turn, as observed with impaired N-glycosylation, could promote its dimerization/aggregation.

## Discussion

Our data suggest that α-α-subunit interactions between Na_v_1.5 proteins observed in heterologous expression system could be due to non-specific mechanisms involving insufficiency of ER folding machinery, and/or non-native intermolecular disulfide bonds and/or hydrophobic interactions.

By using non-reducing SDS-PAGE, we were able to resolve putative Na_v_1.5 dimers in the heterologous expression system represented by tsA201 cells, but not in native mouse cardiomyocytes. These putative Na_v_1.5 dimers were shown to be partially sensitive to the reducing agent, depending on the ER stress and the presence of transmembrane domains. This could signify that the apparent dimers of Na_v_1.5 are likely being represented by the SDS-resistant aggregates, supported by the non-native disulfide bonds.

We show that these dimers/aggregates were unaffected by Na_v_β-subunits but rather relied on the level of *SCN5A* overexpression. We identified that strong interactions occurred between the full-length Na_v_1.5 and its NTD, DI, L1, DII, DIII, and DIV in our cell model.

Our study validates the occurrence of dimerized human VGSC α-subunits, formerly reported by us and other groups^5,7,10–12,14,29,30^. In addition to the previously described homodimerization site of Na_v_1.5 at L1^5^, our group previously observed close proximity between the full-length protein and its truncated NTD^31^. In the present study, we confirm direct interactions between full-length Na_v_1.5 and its NTD, as well as L1. Recently, another inter-channel dimerization of a VGSC α-subunit was reported and suggested to take place through its voltage-sensing domain^17^. In support of these observations, our results show that all four transmembrane domains (i.e., DI, DII, DIII, and DIV) were indeed able to strongly interact with full-length Na_v_1.5.

Interestingly, two-distinct pools of *N*-glycosylated Na_v_1.5 channels were reported for the human embryonic kidney cell line HEK293T (RRID: CVCL_0063), a heterologous expression system highly similar to ours^28^. A previous study proposed the co-existence of fully mature Na_v_1.5 channels that underwent post-*cis*-Golgi glycosylation together with partially mature *N*-glycan-restricted Na_v_1.5 proteins^28^. Even though the latter did not undergo terminal glycosylation, both Na_v_1.5 pools were shown to traffic to the plasma membrane^28^. The authors suggested that the unconventional Golgi-independent pathway of membrane transport could be potentially used for the clearance of Na_v_1.5 proteins accumulating in the ER to prevent ER stress^28^. Similarly, we suggest a possible link between ER stress due to protein overexpression, Na_v_1.5 dimerization/aggregation and its post-translational modifications, such as *N*-glycosylation. According to our results, preventing early *N*-glycosylation of nascent polypeptides with TUN while blocking proteasomal degradation with MG132 tended to promote Na_v_1.5 dimerization/aggregation. We also show that Na_v_1.5 dimers/aggregates reached the plasma membrane. In line with these findings, Förster resonance energy transfer studies demonstrated that Na_v_1.5 α-subunits interacted at the cell surface and that these interactions occurred before protein trafficking to the plasma membrane^5^.

Previously, Na_v_β1 was reported to mediate α-α-subunit interactions between Na_v_1.5 proteins^12^. However, our current data demonstrate that dimerization/aggregation of Na_v_1.5 was unaffected by the presence of Na_v_β1, its alternative splicing form Na_v_β1B, or subunits Na_v_β2, Na_v_β3, and Na_v_β4. This was not surprising to us, as other studies showing oligomerization of human VGSC α-subunits did not use co-expression of Na_v_β-subunits^5,10,11^. In addition, another study concluded that clusterization of Na_v_1.5 is possible with and without Na_v_β3^30^. Unexpectedly, the α-α-subunit interaction site previously described for Na_v_1.5 as Arg493-Arg517 region at L1 was not proven to be specific in our hands^5^. Previously, the authors demonstrated that truncated GFP-tagged constructs Na_v_1.5-Ile450X and Na_v_1.5-Asn470X were unable to co-immunoprecipitate with full-length HA-tagged Na_v_1.5^5^. However, according to our results, Na_v_1.5-Ile450X strongly interacted with full-length Na_v_1.5. Indeed, such an interaction could be mediated via NTD and DI, parts of Na_v_1.5-Ile450X that by themselves were shown to strongly bind to full-length Na_v_1.5 in our current work.

Another discrepancy with our study comes from the previously shown migration of human Na_v_1.5 and Na_v_1.7 as monomers in both reduced and non-reduced states^11^. Based on these observations, the authors argued against the existence of disulfide bonds between α-subunits but not against non-covalent bonds^11^. However, our results indicate that Na_v_1.5 dimers/aggregates were reducing agent-dependent, even though disulfide bonds did not appear to be the primary cause of α-α-subunit interactions. One possible explanation could be technical: we used Tris-acetate SDS-PAGE in contrast to SDS-urea-PAGE used in previous work. As a strong protein denaturant, urea might assist in protein unfolding and/or stabilization of the unfolded state. If this is the case and non-native disulfide bridges appear during partial protein refolding as a result of our experimental procedures, then it would not explain the absence of Na_v_1.5 dimers/aggregates in native cardiac tissues/cells when using the same non-reducing Tris-acetate SDS-PAGE protocol. Interestingly, for the 30 proteins analyzed, no correlation was found between the extent of urea-induced protein unfolding and the presence/absence of disulfide bridges in the native form^32^. Additionally, another explanation could be experimental: cellular machinery providing for post-translational modifications, including glycosylation, is significantly different between the heterologous expression system used in this study, human tsA201 cells, and *Xenopus laevis* oocytes used in previous work^11^.

Importantly, most of our data were obtained in a heterologous expression system using a protein overexpression approach. Thus, it is possible that the observed interactions and effects on Na_v_1.5 dimerization/aggregation were largely affected by non-native ectopic gene expression. Furthermore, we cannot exclude possibility that the overexpression of truncated constructs with full-length Na_v_1.5 may inherently favor nonspecific interactions.

Another limitation of our study is that by using non-reducing Tris-acetate SDS-PAGE, we were only able to resolve putative Na_v_1.5 dimers that primarily depended on the disulfide bonds. Therefore, whenever possible, we performed co-immunoprecipitation analysis as an additional confirmation of the α-α-subunit interactions.

Biochemical interactions between α-α-subunits of Na_v_1.5 were largely used to describe/explain the dominant-negative effect (DNE) of *SCN5A* (reviewed in^26^). DNE was shown for both native and heterologous cell models^33–35^. Accordingly, it was hypothesized that DNE occurred due to direct interactions between wild-type and mutant Na_v_1.5 because the trafficking, turnover, and function of the interacting pair would be largely affected^36^. However, a direct link between DNE and Na_v_1.5 has yet to be shown, as disruption of the protein-protein interaction between mutant and wild-type channels should lead to the rescue of *I*_Na_.

Our data suggest a link between Na_v_1.5 dimerization/aggregation and ER stress. Although we did not detect Na_v_1.5 dimers in non-diseased mouse cardiomyocytes, such α-α-subunits interactions may occur after ER stress induction. Indeed, fever, pharmacological agents, and age might promote ER stress and are known risk factors for the manifestation of Brugada syndrome, a cardiac disease primarily attributed to *SCN5A* loss-of-function variants^35^.

## Conclusion

Taken together, our data emphasize the importance of controlling exogenous cDNA amount and raise caution regarding challenges related to investigating the oligomerization of Na_v_1.5 in a heterologous expression system. Therefore, further work needs to be carried out, specifically, using an in vivo model where both alleles of *SCN5A* are genetically modified to produce native Na_v_1.5 proteins with different tags.

### Supporting information

Supporting information Figure S1 with its legend shows the data to complement Figure 1. 

## Electronic Supplementary Material

Below is the link to the electronic supplementary material.

Supplementary Material 1

Supplementary Material 2

Supplementary Material 3

Supplementary Material 4

Supplementary Material 5

Supplementary Material 6

Supplementary Material 7

Supplementary Material 8

Supplementary Material 9

Supplementary Material 10

Supplementary Material 11

Supplementary Material 12

Supplementary Material 13

Supplementary Material 14

Supplementary Material 15

## Data Availability

Data are available in the article itself and its supplementary materials.

## Abbreviations

CMV: cytomegalovirus
CTD: C-terminal domain
DNE: dominant-negative effect
DTT: dithiothreitol
HA: haemagglutinin
I_Na_: Na^+^ current
LDS: lithium dodecyl sulfate
Na^+^: sodium ion
Na_v_1.x: α-subunit of the voltage-gated sodium channel
Na_v_β: β-subunit of the voltage-gated sodium channel
NTD: N-terminal domain
PBS: Phosphate-buffered saline
SDS-PAGE: sodium dodecyl sulfate-polyacrylamide gel electrophoresis
TBS: Tris-buffered saline
TUN: tunicamycin
UbC: ubiquitin C
UPR: unfolded protein response
VGSC: voltage-gated sodium channel
