# Supplementary material for: Biochemical assessment of α-α-subunit interactions of Nav1.5 in a heterologous expression system
Source: Sci Rep. 2026 May 4;16:20583. doi: 10.1038/s41598-026-50463-9 (PMC13333962; doi:10.1038/s41598-026-50463-9)
Supplement: Supplementary file 1 — Supplementary Material 1 [file 41598_2026_50463_MOESM1_ESM.pdf]

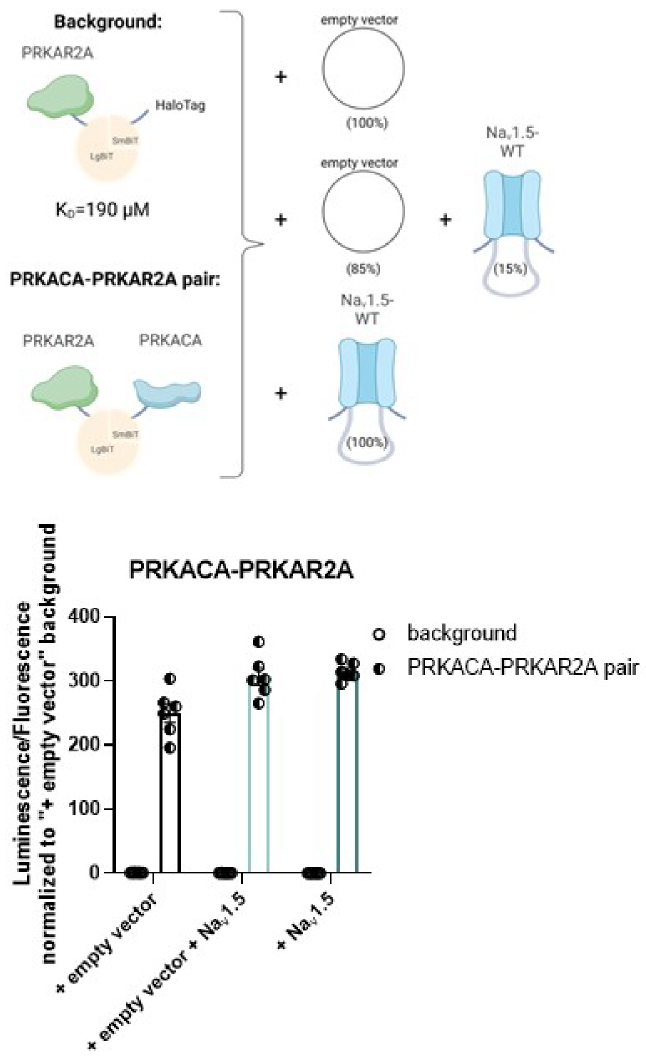

Figure S1. **Na$_V$1.5 does not affect the interaction between proteins of the control pair represented by PRKACA-PRKAR2A.** Schematic illustration of the NanoBiT assay, when co-expressing different quantities of untagged Na$_V$1.5 together with the control pair of PRKAR2A-LgBiT and PRKACA-SmBiT. Background signal was determined by co-expression of PRKAR2A-LgBiT with a non-interacting control, HaloTag-SmBiT. Results of the NanoBiT assay are presented as the relative intensity of luminescence (indicating the level of protein-protein interactions) normalized to the fluorescence (indicating the number of cells) of tsA201 cells 48 hours after transfection. Each dataset was normalized to "+ empty vector" control. Data are presented as mean ± SD from six technical replicates.
