# Supplementary material for: Biochemical assessment of α-α-subunit interactions of Nav1.5 in a heterologous expression system
Source: Sci Rep. 2026 May 4;16:20583. doi: 10.1038/s41598-026-50463-9 (PMC13333962; doi:10.1038/s41598-026-50463-9)
Supplement: Supplementary file 3 — Supplementary Material 3 [file 41598_2026_50463_MOESM3_ESM.docx]

**Table S1. Description of cDNA constructs used in this study.**

| cDNA construct | Transcript (Genbank) | Plasmid size, kb, and expected molecular weight, kDa | Source |
| --- | --- | --- | --- |
| pcDNA3.1-CMV | Empty vector | 5.8 kb | V86020, Invitrogen (USA) |
| pMB1-SV40 |  | 1.9 kb | a gift from Jordan Green (Addgene plasmid # 127639; http://n2t.net/addgene:127639; RRID:Addgene_127639) (USA) |
| pCAGEN-UbC |  | 3.1 kb | a gift from Connie Cepko (Addgene plasmid # 11155; http://n2t.net/addgene:11155; RRID:Addgene_11155) ^20^ |
| pcDNA3.1-CMV-*SCN5A*-WT | NM_000335.5 | 11.9 kb, 230 kDa | GenScript, NJ, USA |
| pMB1-SV40-*SCN5A*-WT |  | 8 kb, 230 kDa |  |
| pCAGEN-UbC-*SCN5A*-WT |  | 9.2 kb, 230 kDa |  |
| pcDNA3.1-3xFLAG(Nter)-*SCN5A*-WT |  | 12 kb, 230 kDa |  |
| pcDNA3.1-3xHA(Nter)-*SCN5A*-WT |  | 12 kb, 230 kDa |  |
| pcDNA3.1-1xmEGFP(Nter)-*SCN5A*-WT |  | 12.7 kb, 250 kDa |  |
| pFN217K-LgBiT(Nter)-*SCN5A*-WT |  | 11.4 kb, 248 kDa | Cloned from pcDNA3.1-CMV-*SCN5A*-WT into NanoBiT^®^ CMV Flexi BiBit ready vectors (ID# CS1603B33) according to Flexi^®^ vector systems technical manual #TM254 from Promega (Switzerland), as previously described ^12^ |
| pFC219K-*SCN5A*-WT-LgBiT(Cter) |  | 11.3 kb, 248 kDa |  |
| pFN218K-SmBiT(Nter)-*SCN5A*-WT |  | 10.8 kb, 232 kDa |  |
| pFC220K-*SCN5A*-WT-SmBiT(Cter) |  | 10.8 kb, 232 kDa |  |
| pF5K-*SCN5A*-WT |  | 10.7 kb, 230 kDa |  |
| CMV/SmBiT-CA/BlastR | NM_002730.4 | 5.4 kb | NanoBiT^®^ CMV PPI control vectors (ID# CS1603B54) from Promega (Switzerland) |
| CMV/LgBiT-R2A/HygR | NM_004157.4 | 6 kb |  |
| BiBiT-RI/SmBiT-CA/LgBiT-R2A/BlastR | NM_002730.4 and NM_004157.4 | 7.3 kb |  |
| CMV/HaloTag^®^-SmBiT | HM157289.1 | 5.2 kb, 35 kDa |  |
| 1xGFP(Cter)-Tpr | NM_003292.3 | 11.7 kb, 267 kDa | a gift from Larry Gerace (Addgene plasmid # 35024; http://n2t.net/addgene:35024; RRID:Addgene_35024) ^21^ |
| pCMV6-*SCN1BB* | NM_199037.5 | 5.7 kb, 30 kDa | #RC222161, Origene (USA) |
| pCIH-*SCN1B* | NM_001037.5 | 8 kb, 25 kDa | a gift from Simon Tate |
| pCIH-*SCN2B* | NM_004588.5 | 8 kb, 25 kDa |  |
| pFBM-*SCN3B* | NM_018400.4 | 8 kb, 25 kDa |  |
| pCIH-*SCN4B* | NM_174934.4 | 8 kb, 30 kDa |  |
| pcDNA3.1-3xFLAG(Nter)-*SCN5A*-I450X | truncated NM_000335.5, stop codon after Ile450 | 7.2 kb, 60 kDa | GenScript, NJ, USA |
| pcDNA3.1-3xFLAG(Nter)-*SCN5A*-I450X-allCys-mut | same as previous with Cys139Ala, Csy145Ala, Cys182Ala, Cys260Ala, Cys280Ala, Cys326Ala, Cys335Ala, Cys341Ala, Cys373Ala | 7.2 kb, 60 kDa |  |
| pcDNA3.1-1xFLAG(Nter)-*SCN5A*-R535X | truncated NM_000335.5, stop codon after Arg535 | 7.5 kb, 62 kDa |  |
| pcDNA3.1-3xFLAG(Nter)-*SCN5A*-R535X-extraCys-mut | same as previous with Cys280Ala, Cys326Ala, Cys335Ala, Cys341Ala | 7.6 kb, 67 kDa |  |
| pcDNA3.1-3xFLAG(Nter)-*SCN5A*-R535X-extraCys-mut | same as previous with Cys139Ala, Cys145Ala, Cys182Ala, Cys260Ala, Cys280Ala, Cys326Ala, Cys335Ala, Cys341Ala, Cys373Ala | 7.6 kb, 67 kDa |  |
| pcDNA3.1-3xHA(Nter)-*SCN5A*-NTD | MYPYDVPDYAGYPYDVPDYAGYPYDVPDYAGGGGSGGGGSGGGGSANFLLPRGTSSFRRFTRESLAAIEKRMAEKQARGSTTLQESREGLPEEEAPRPQLDLQASKKLPDLYGNPPQELIGEPLEDLDPFYSTQKTFIVLNKGKTIFRFSATNALYVLSPFHPIRRAAVKILVHS* | 6.3 kb, 17 kDa |  |
| pcDNA3.1-3xHA(Nter)-*SCN5A*-DI | MYPYDVPDYAGYPYDVPDYAGYPYDVPDYAGGGGSGGGGSGGGGSLFNMLIMCTILTNCVFMAQHDPPPWTKYVEYTFTAIYTFESLVKILARGFCLHAFTFLRDPWNWLDFSVIIMAYTTEFVDLGNVSALRTFRVLRALKTISVISGLKTIVGALIQS  VKKLADVMVLTVFCLSVFALIGLQLFMGNLRHKCVRNFTALNGTNGSVEADGLVWESLDLYLSDPENYLLKNGTSDVLLC  GNSSDAGTCPEGYRCLKAGENPDHGYTSFDSFAWAFLALFRLMTQDCWERLYQQTLRSAGKIYMIFFMLVIFLGSFYLVN  LILA* | 6.8 kb, 36 kDa |  |
| pcDNA3.1-3xHA(Nter)-*SCN5A*-L1 | MYPYDVPDYAGYPYDVPDYAGYPYDVPDYAGGGGSGGGGSGGGGSVVAMAYEEQNQATIAETEEKEKRFQEAMEMLKKEHEALTIRGVDTVSRSSLEMSPLAPVNSHERRSKRRKRMSSGTEECGEDRLPKSDSEDGPRAMNHLSLTRGLSRTSMKPRSSRGSIFTFRRRDLGSEADFADDENSTAGESESHHTSLLVPWPLRRTSAQGQPSPGTSAPGHALHGKKNSTVDCNGVVSLLGAGDPEATSPGSHLLRPVMLEHPPDTTTPSEEPGGPQMLTSQAPCVDGFEEPGARQRALSAVSVLTSALEELEESRHKCPPCWNRLAQRYLIWECCPLWMSIKQGVKLVVMDP* | 7.1 kb, 48 kDa |  |
| pcDNA3.1-3xHA(Nter)-*SCN5A*-DII | MYPYDVPDYAGYPYDVPDYAGYPYDVPDYAGGGGSGGGGSGGGGSFTDLTITMCIVLNTLFMALEHYNMTSEFEEMLQVGNLVFTGIFTAEMTFKIIALDPYYYFQQGWNIFDSIIVILSLMELGLSRMSNLSVLRSFRLLRVFKLAKSWPTLNTLIKIIGNSVGALGNLTLVLAIIVFIFAVVGMQLFGKNYSELRDSDSGLLPRWHMMDFFHAFLIIFRILCGEWIETMWDCMEVSGQSLCLLVFLLVMVIGNLVVLNLFLALL* | 6.8 kb, 36 kDa |  |
| pcDNA3.1-3xHA(Nter)-*SCN5A*-L2 | MYPYDVPDYAGYPYDVPDYAGYPYDVPDYAGGGGSGGGGSGGGGSLSSFSADNLTAPDEDREMNNLQLALARIQRGLRFVKRTTWDFCCGLLRQRPQKPAALAAQGQLPSCIATPYSPPPPETEKVPPTRKETRFEEGEQPGQGTPGDPEPVCVPIAVAESDTDDQEEDEENSLGTEEESSKQESQPVSGGPEAPPDSRTWSQVSATASSEAEASASQADWRQQWKAEPQAPGCGETPEDSCSEGSTADMTNTAELLEQIPDLGQDVKDPEDCFTEGCVRRCPCCAVDTTQAPGKVWWRLRKTCYHIVEHSW* | 6.9 kb, 41 kDa |  |
| pcDNA3.1-3xHA(Nter)-*SCN5A*-DIII | MYPYDVPDYAGYPYDVPDYAGYPYDVPDYAGGGGSGGGGSGGGGSFETFIIFMILLSSGALAFEDIYLEERKTIKVLLEYADKMFTYVFVLEMLLKWVAYGFKKYFTNAWCWLDFLIVDVSLVSLVANTLGFAEMGPIKSLRTLRALRPLRALSRFEGMRVVVNALVGAIPSIMNVLLVCLIFWLIFSIMGVNLFAGKFGRCINQTEGDLPLNYTIVNNKSQCESLNLTGELYWTKVKVNFDNVGAGYLALLQVATFKGWMDIMYAAVDSRGYEEQPQWEYNLYMYIYFVIFIIFGSFFTLNLFI* | 6.8 kb, 36 kDa |  |
| pcDNA3.1-3xHA(Nter)-*SCN5A*-L3 | MYPYDVPDYAGYPYDVPDYAGYPYDVPDYAGGGGSGGGGSGGGGSGVIIDNFNQQKKKLGGQDIFMTEEQKKYYNAMKKLGSKKPQKPIPRPLNKYQGFIFDIVTKQA* | 6.3 kb, 17 kDa |  |
| pcDNA3.1-3xHA(Nter)-*SCN5A*-DIV | MYPYDVPDYAGYPYDVPDYAGYPYDVPDYAGGGGSGGGGSGGGGSFDVTIMFLICLNMVTMMVETDDQSPEKINILAKINLLFVAIFTGECIVKLAALRHYYFTNSWNIFDFVVVILSIVGTVLSDIIQKYFFSPTLFRVIRLARIGRILRLIRGAKGIRTLLFALMMSLPALFNIGLLLFLVMFIYSIFGMANFAYVKWEAGIDDMFNFQTFANSMLCLFQITTSAGWDGLLSPILNTGPPYCDPTLPNSNGSRGDCGSPAVGILFFTTYIIISFLIVVNMYIAII* | 6.8 kb, 36 kDa |  |
| pcDNA3.1-3xHA(Nter)-*SCN5A*-NTD | MYPYDVPDYAGYPYDVPDYAGYPYDVPDYAGGGGSGGGGSGGGGSLENFSVATEESTEPLSEDDFDMFYEIWEKFDPEATQFIEYSVLSDFADALSEPLRIAKPNQISLINMDLPMVSGDRIHCMDILFAFTKRVLGESGEMDALKIQMEEKFMAANPSKISYEPITTTLRRKHEEVSAMVIQRAFRRHLLQRSLKHASFLFRQQAGSGLSEEDAPEREGLIAYVMSENFSRPLGPPSSSSISSTSFPPSYDSVTRATSDNLQVRGSDYSHSEDLADFPPSPDRDRESIV* | 7 kb, 38 kDa |  |
| pUC19-m*Scn5a*-WT | NM_001253860.1 | 8.7 kb, 225 kDa | a gift from Thomas Zimmer |
| pcDNA3.1-m*Scn5a*-WT |  | 11.8 kb, 225 kDa |  |
