# Supplementary material for: Biochemical assessment of α-α-subunit interactions of Nav1.5 in a heterologous expression system
Source: Sci Rep. 2026 May 4;16:20583. doi: 10.1038/s41598-026-50463-9 (PMC13333962; doi:10.1038/s41598-026-50463-9)
Supplement: Supplementary file 5 — Supplementary Material 5 [file 41598_2026_50463_MOESM5_ESM.pdf]

Fig1A

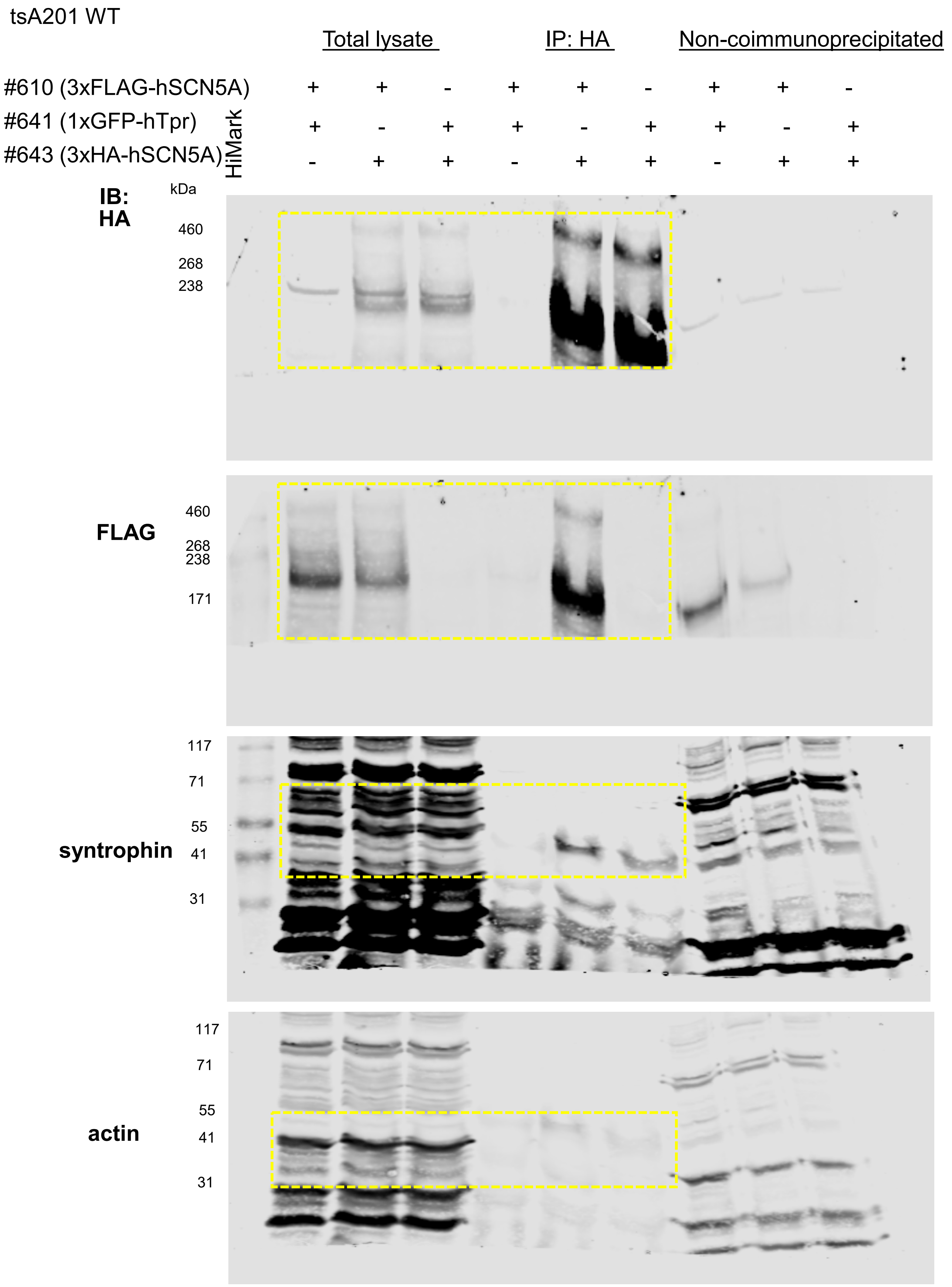
tsA201 WT
Total lysate
IP: HA
Non-coimmunoprecipitated
#610 (3xFLAG-hSCN5A) + + - + + - + + -
#641 (1xGFP-hTpr) + - + + - + + - +
#643 (3xHA-hSCN5A) - + + - + + - + +
HiMark
IB: HA
kDa
460
268
238
FLAG
460
268
238
171
syntrophin
117
71
55
41
31
actin
117
71
55
41
31

Fig1D

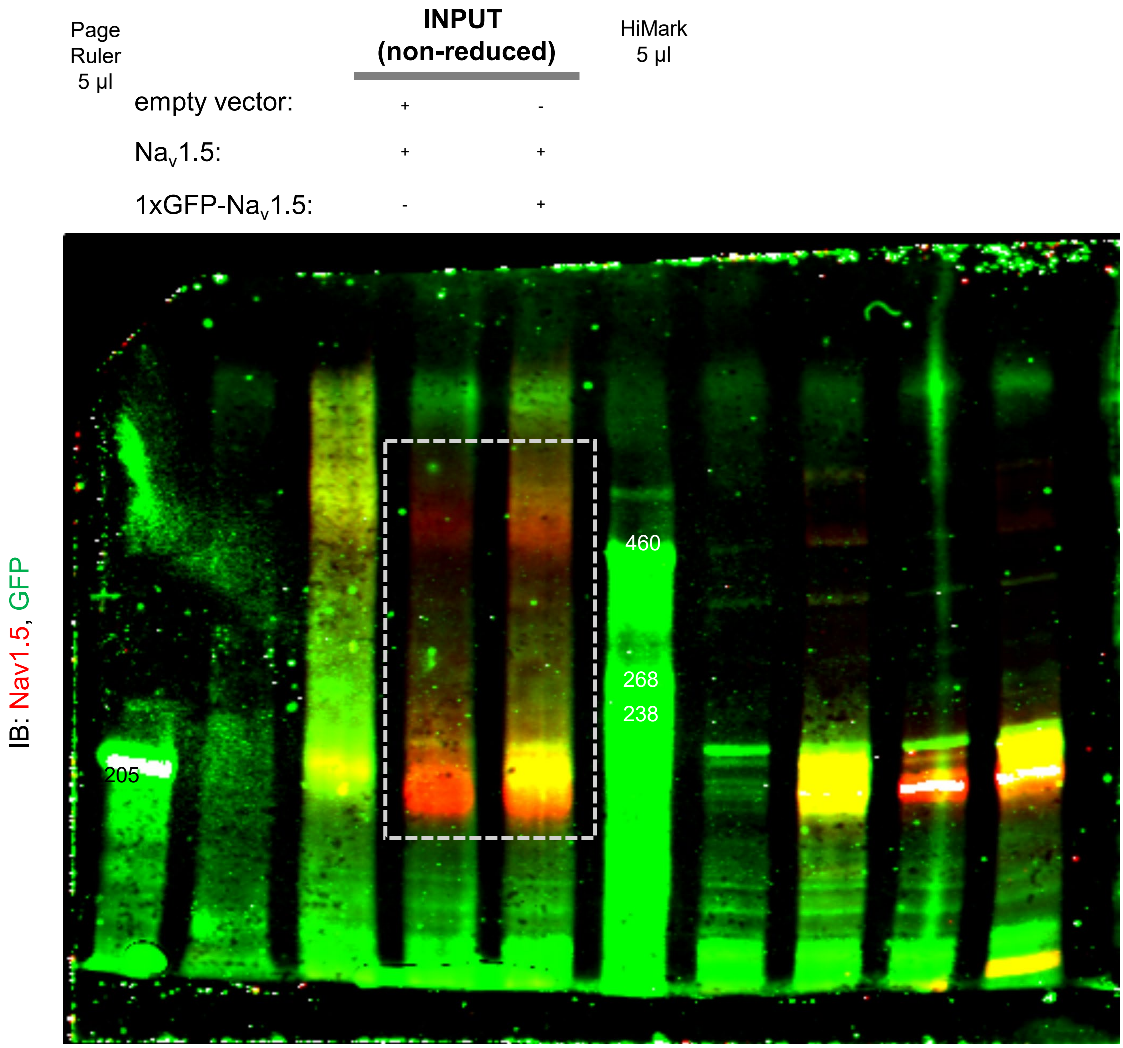
Page Ruler 5 µl
INPUT (non-reduced)
HiMark 5 µl
empty vector: + -
Na$_v$1.5: + +
1xGFP-Na$_v$1.5: - +
IB: Nav1.5, GFP
460
268
238
205

Fig1E

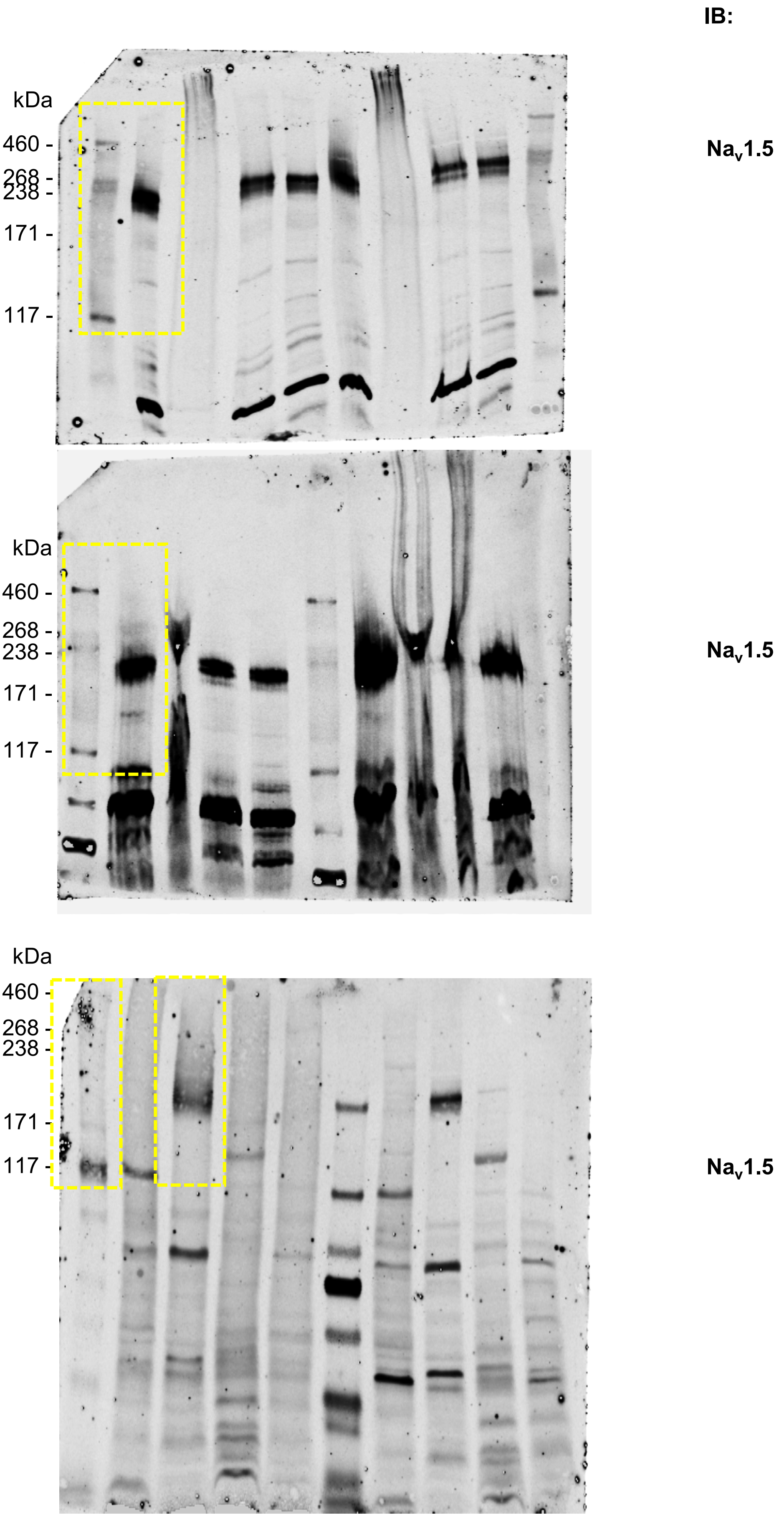
IB:
kDa
460 -
268 -
238 -
171 -
117 -
$Na_v1.5$
kDa
460 -
268 -
238 -
171 -
117 -
$Na_v1.5$
kDa
460 -
268 -
238 -
171 -
117 -
$Na_v1.5$

Fig1F

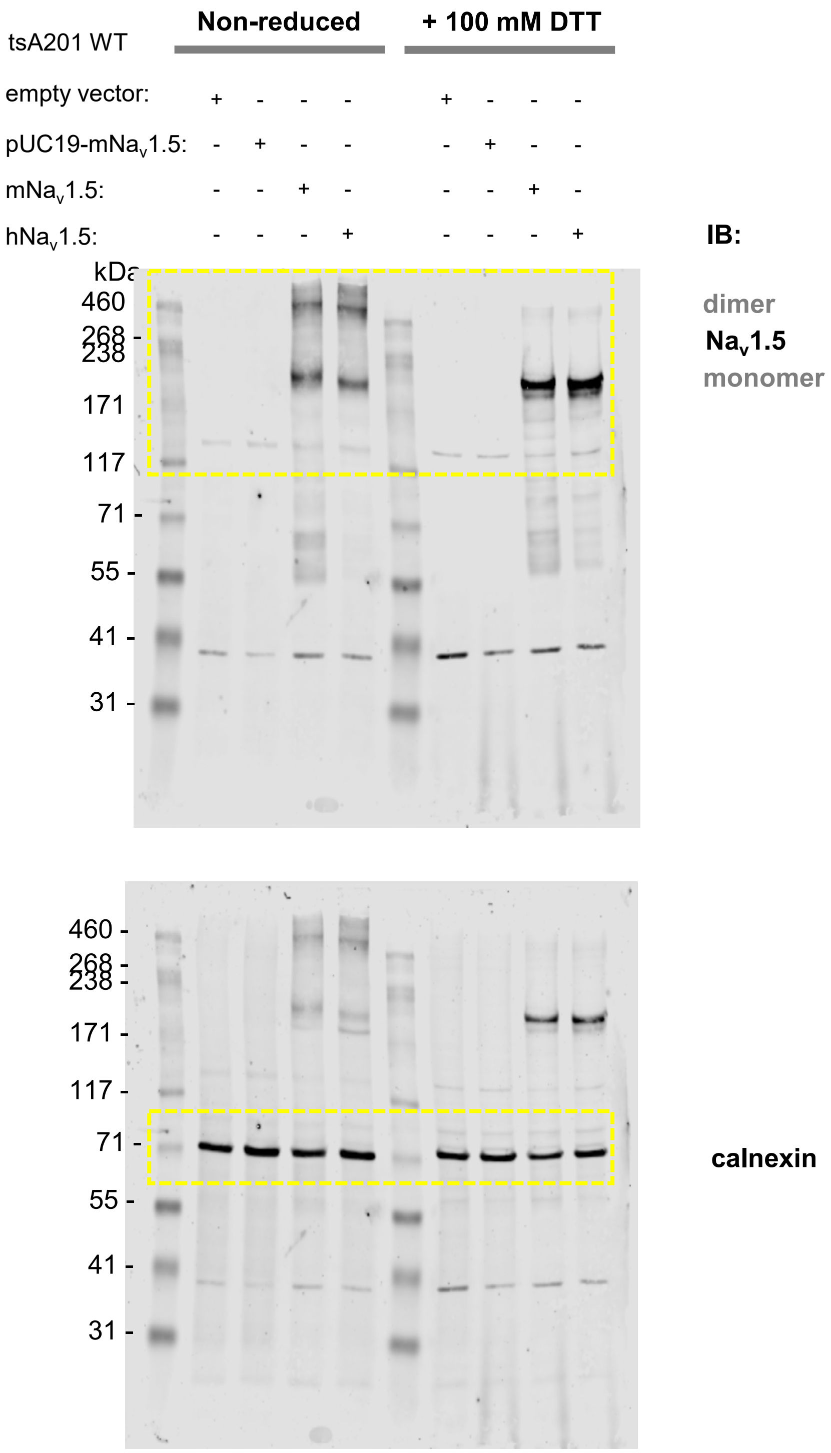

tsA201 WT
Non-reduced
+ 100 mM DTT
empty vector: + - - - + - - -
pUC19-mNav1.5: - + - - - + - -
mNav1.5: - - + - - - + -
hNav1.5: - - - + - - - +
IB:
kDa
460
268
238
171
117
71
55
41
31
dimer
Nav1.5
monomer
460
268
238
171
117
71
55
41
31
calnexin
