# Supplementary material for: Biochemical assessment of α-α-subunit interactions of Nav1.5 in a heterologous expression system
Source: Sci Rep. 2026 May 4;16:20583. doi: 10.1038/s41598-026-50463-9 (PMC13333962; doi:10.1038/s41598-026-50463-9)

Fig2A

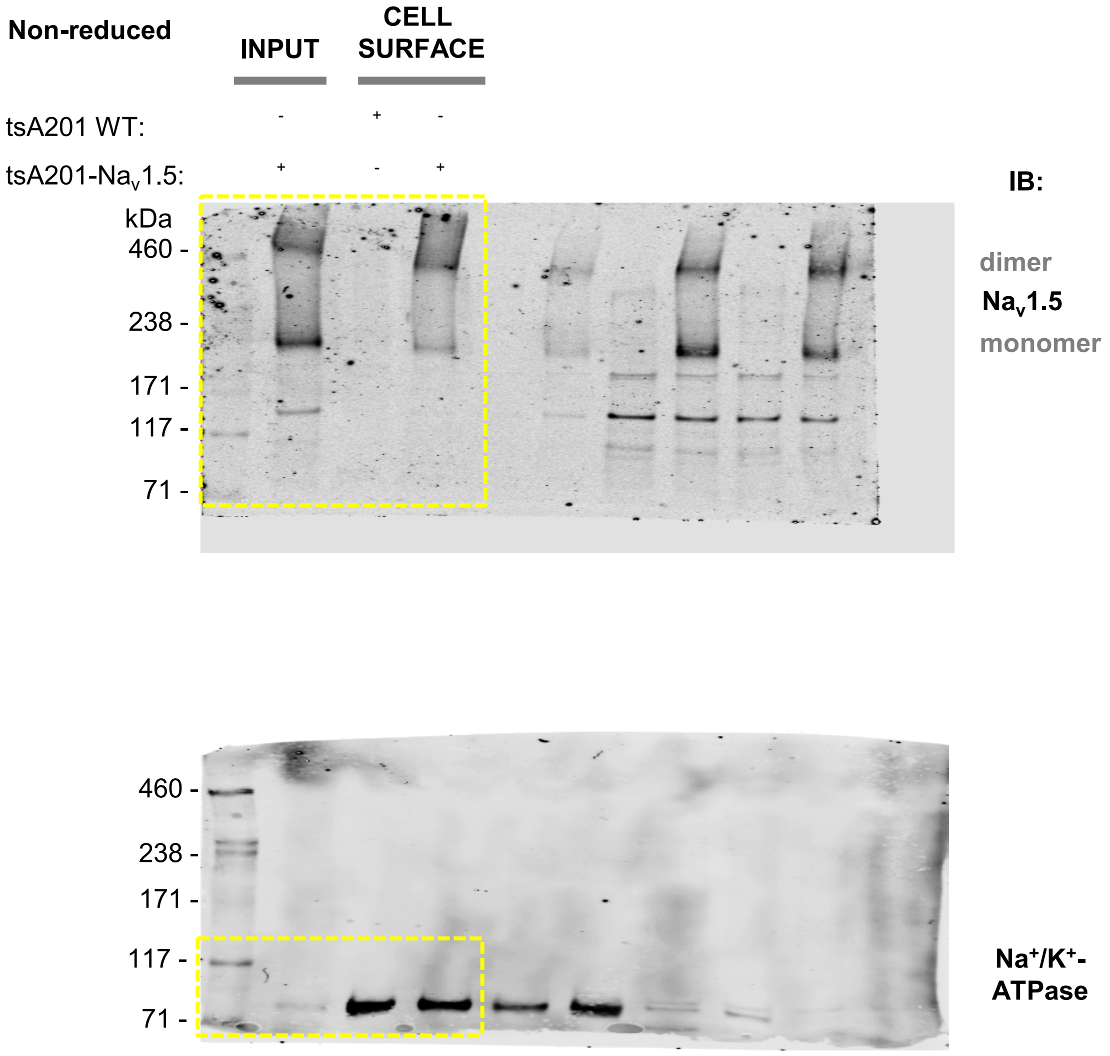
Non-reduced
INPUT
CELL SURFACE
tsA201 WT: - + -
tsA201-Na$_v$1.5: + - +
IB:
kDa
460 -
238 -
171 -
117 -
71 -
dimer
Na$_v$1.5
monomer
460 -
238 -
171 -
117 -
71 -
Na$^+$/K$^+$-ATPase

Fig2C

tsA201 WT

	INPUT (+100 mM DTT)						IP: HA (+100 mM DTT)					
3xHA-Na$_v$1.5:	+	+	+	+	+	+	+	+	+	+	+	+
Na$_v$1.5:	+	-	-	-	-	-	+	-	-	-	-	-
1XFLAG-Na$_v$1.5-R535X:	-	+	-	-	-	-	-	+	-	-	-	-
3XFLAG-Na$_v$1.5-R535X-extraCys-mut:	-	-	+	-	-	-	-	-	+	-	-	-
3XFLAG-Na$_v$1.5-R535X-allCys-mut:	-	-	-	+	-	-	-	-	-	+	-	-
3XFLAG-Na$_v$1.5-I450X:	-	-	-	-	+	-	-	-	-	-	+	-
3XFLAG-Na$_v$1.5-I450X-allCys-mut:	-	-	-	-	-	+	-	-	-	-	-	+

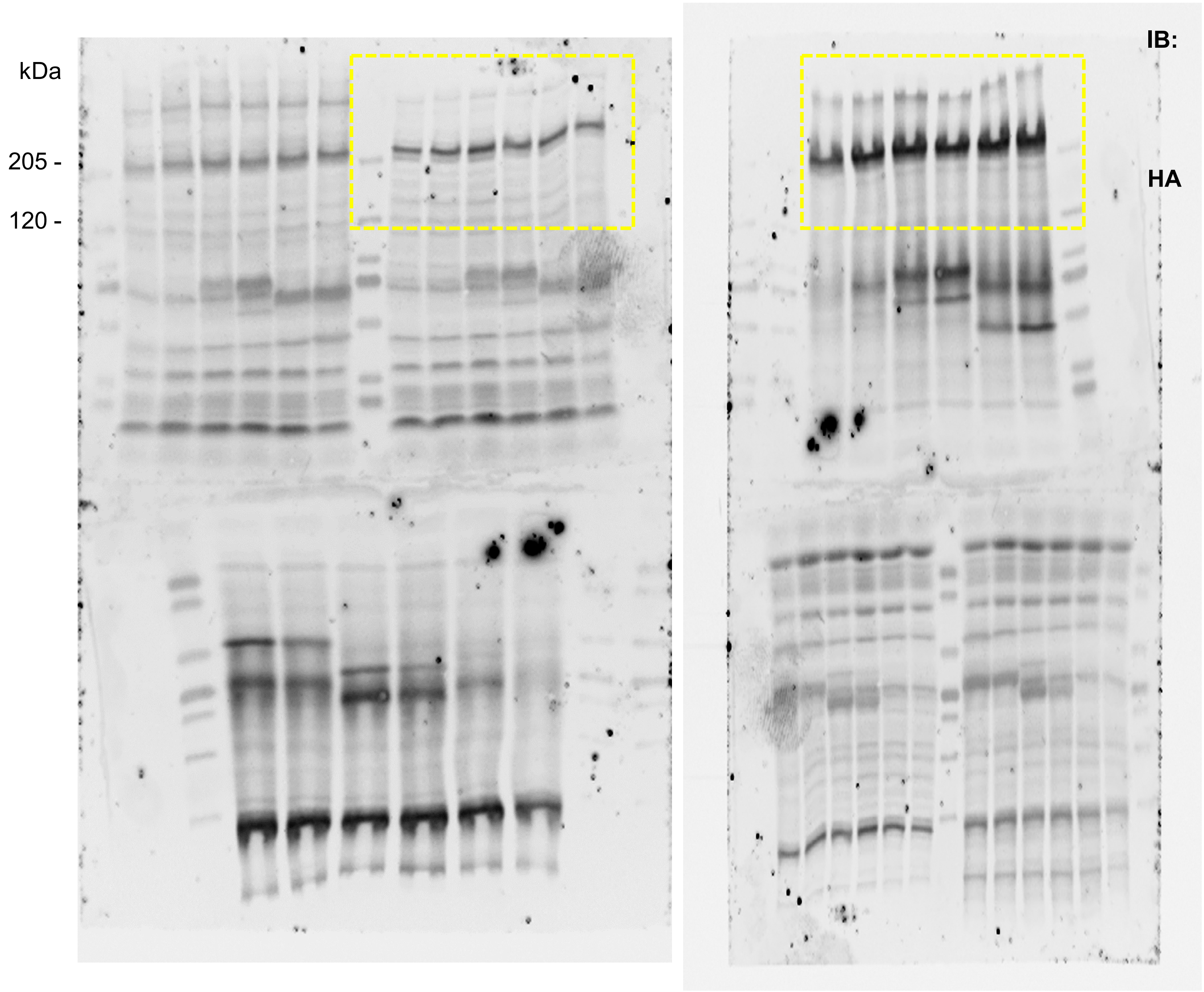

Fig2C

tsA201 WT	INPUT (+100 mM DTT)						IP: HA (+100 mM DTT)					
3xHA-Na_v1.5:	+	+	+	+	+	+	+	+	+	+	+	+
Na_v1.5:	+	-	-	-	-	-	+	-	-	-	-	-
1XFLAG-Na_v1.5-R535X:	-	+	-	-	-	-	-	+	-	-	-	-
3XFLAG-Na_v1.5-R535X-extraCys-mut:	-	-	+	-	-	-	-	-	+	-	-	-
3XFLAG-Na_v1.5-R535X-allCys-mut:	-	-	-	+	-	-	-	-	-	+	-	-
3XFLAG-Na_v1.5-I450X:	-	-	-	-	+	-	-	-	-	-	+	-
3XFLAG-Na_v1.5-I450X-allCys-mut:	-	-	-	-	-	+	-	-	-	-	-	+

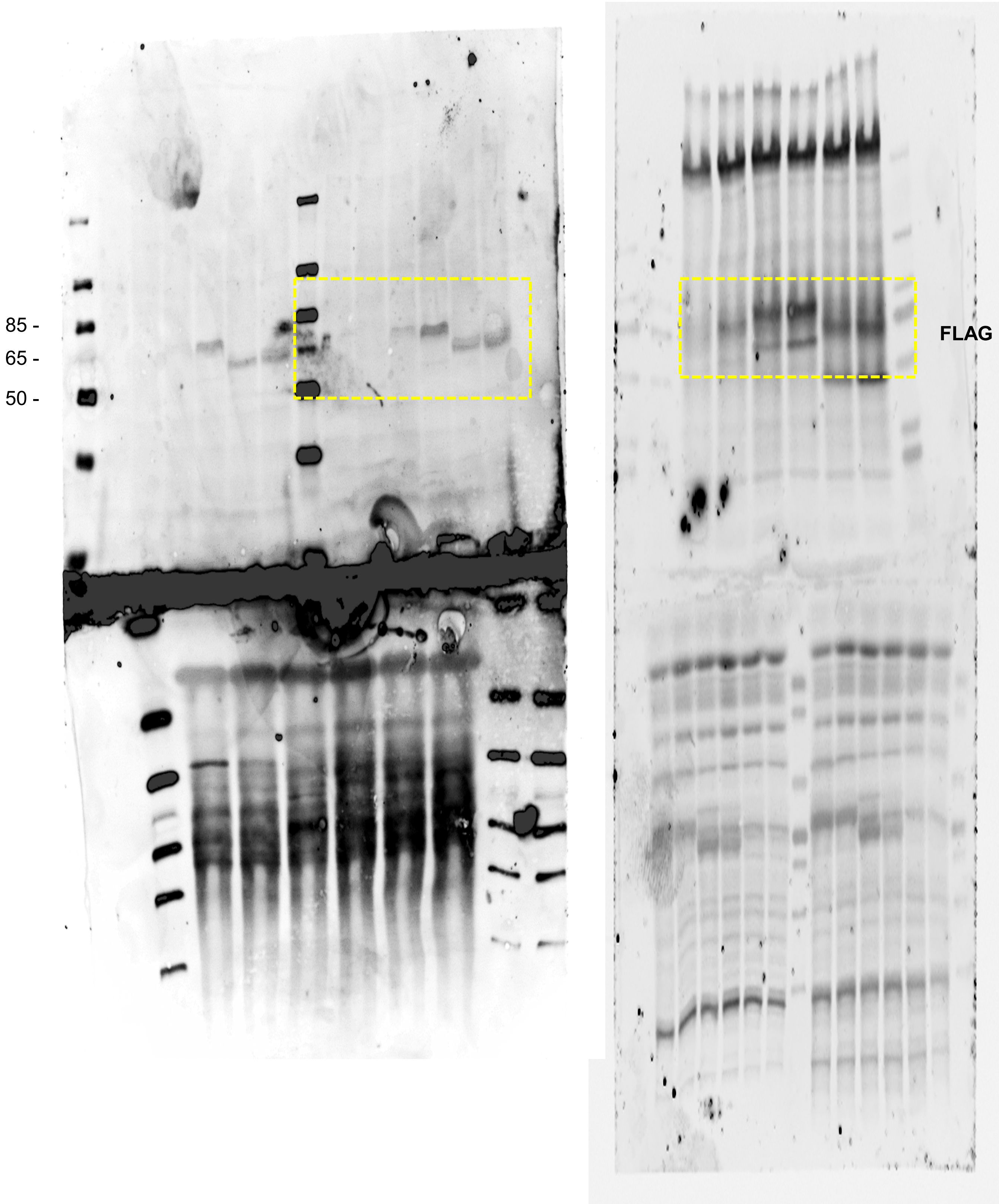

Fig2C

tsA201 WT

	INPUT (+100 mM DTT)						IP: HA (+100 mM DTT)					
3xHA-Na_v1.5:	+	+	+	+	+	+	+	+	+	+	+	+
Na_v1.5:	+	-	-	-	-	-	+	-	-	-	-	-
1XFLAG-Na_v1.5-R535X:	-	+	-	-	-	-	-	+	-	-	-	-
3XFLAG-Na_v1.5-R535X-extraCys-mut:	-	-	+	-	-	-	-	-	+	-	-	-
3XFLAG-Na_v1.5-R535X-allCys-mut:	-	-	-	+	-	-	-	-	-	+	-	-
3XFLAG-Na_v1.5-I450X:	-	-	-	-	+	-	-	-	-	-	+	-
3XFLAG-Na_v1.5-I450X-allCys-mut:	-	-	-	-	-	+	-	-	-	-	-	+

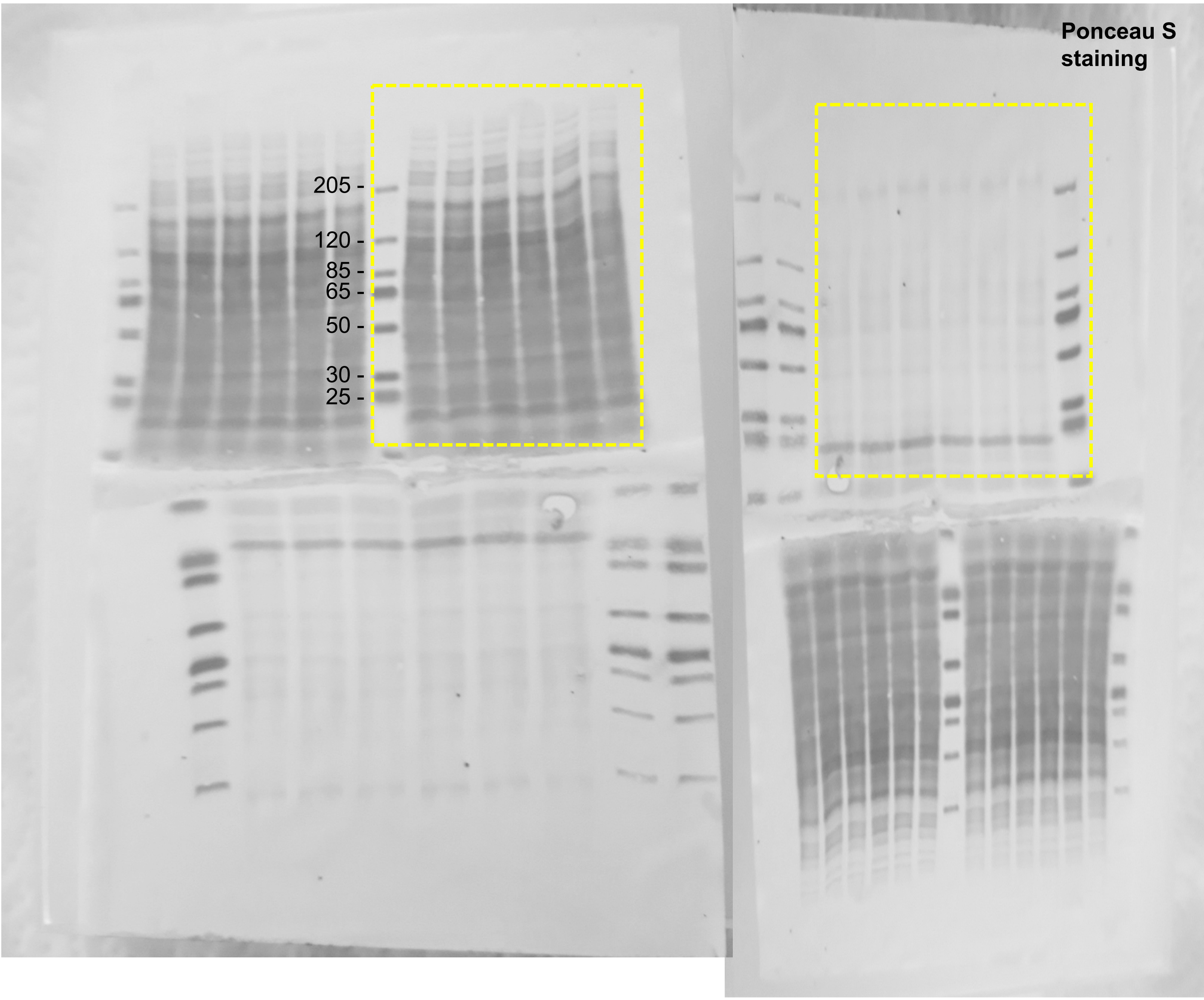

Supplement: Supplementary file 6 — Supplementary Material 6 [file 41598_2026_50463_MOESM6_ESM.pdf]
