# Supplementary material for: Biochemical assessment of α-α-subunit interactions of Nav1.5 in a heterologous expression system
Source: Sci Rep. 2026 May 4;16:20583. doi: 10.1038/s41598-026-50463-9 (PMC13333962; doi:10.1038/s41598-026-50463-9)

Fig3A (representative blot taken into analysis in Fig3C)

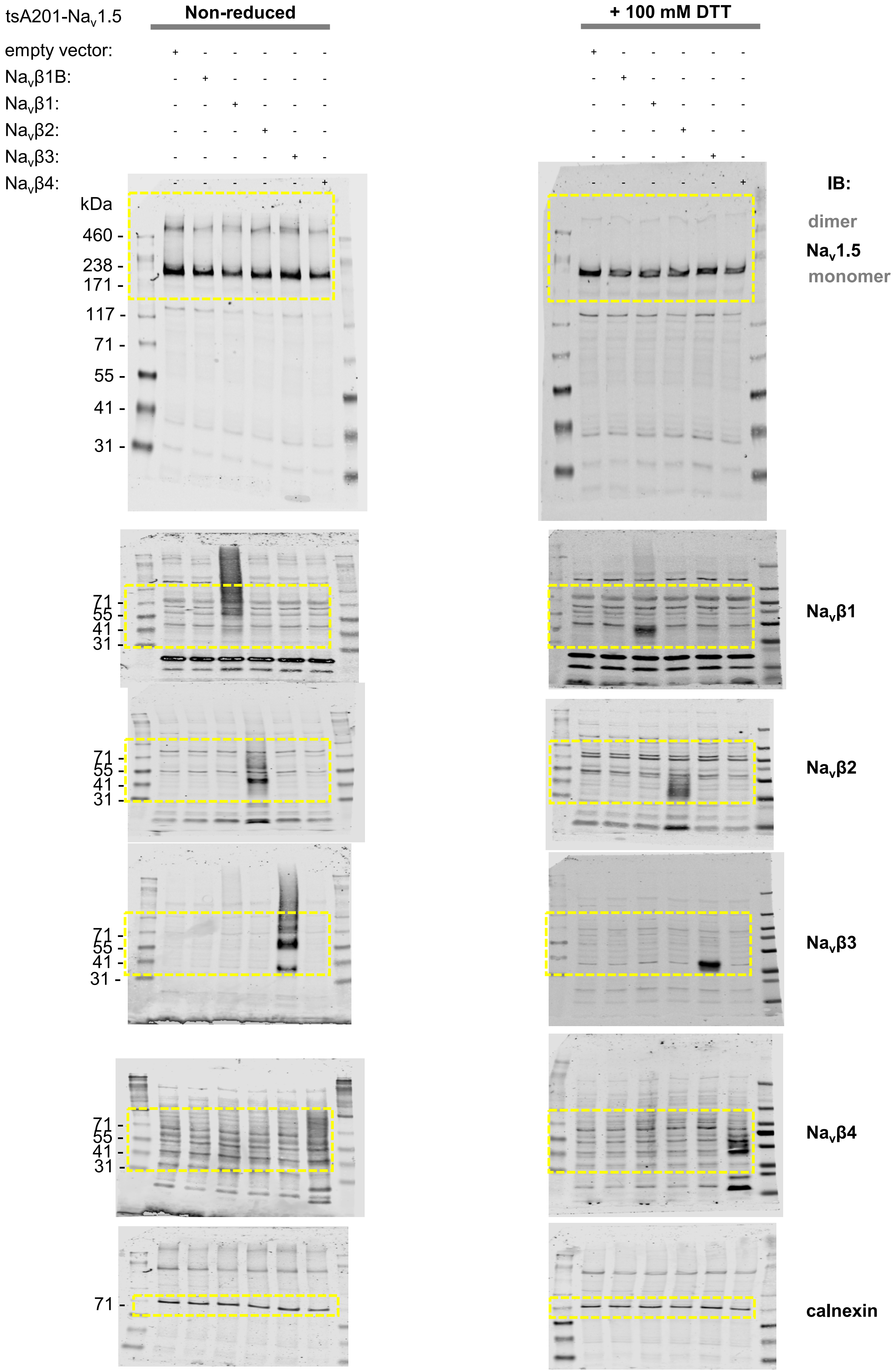

Fig3A (additional repeat taken into analysis in Fig3C)

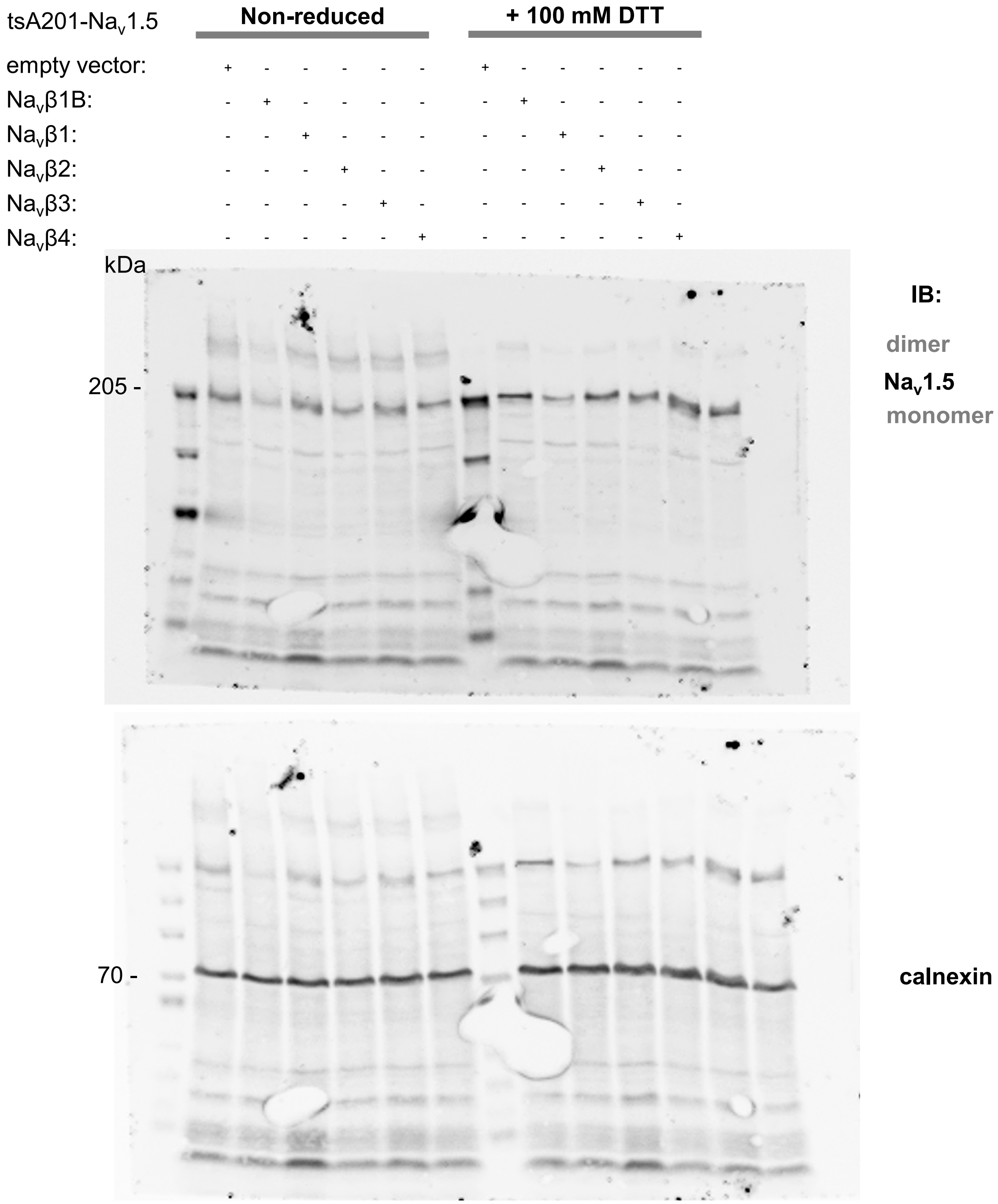

Fig3A (additional repeat taken into analysis in Fig3C)

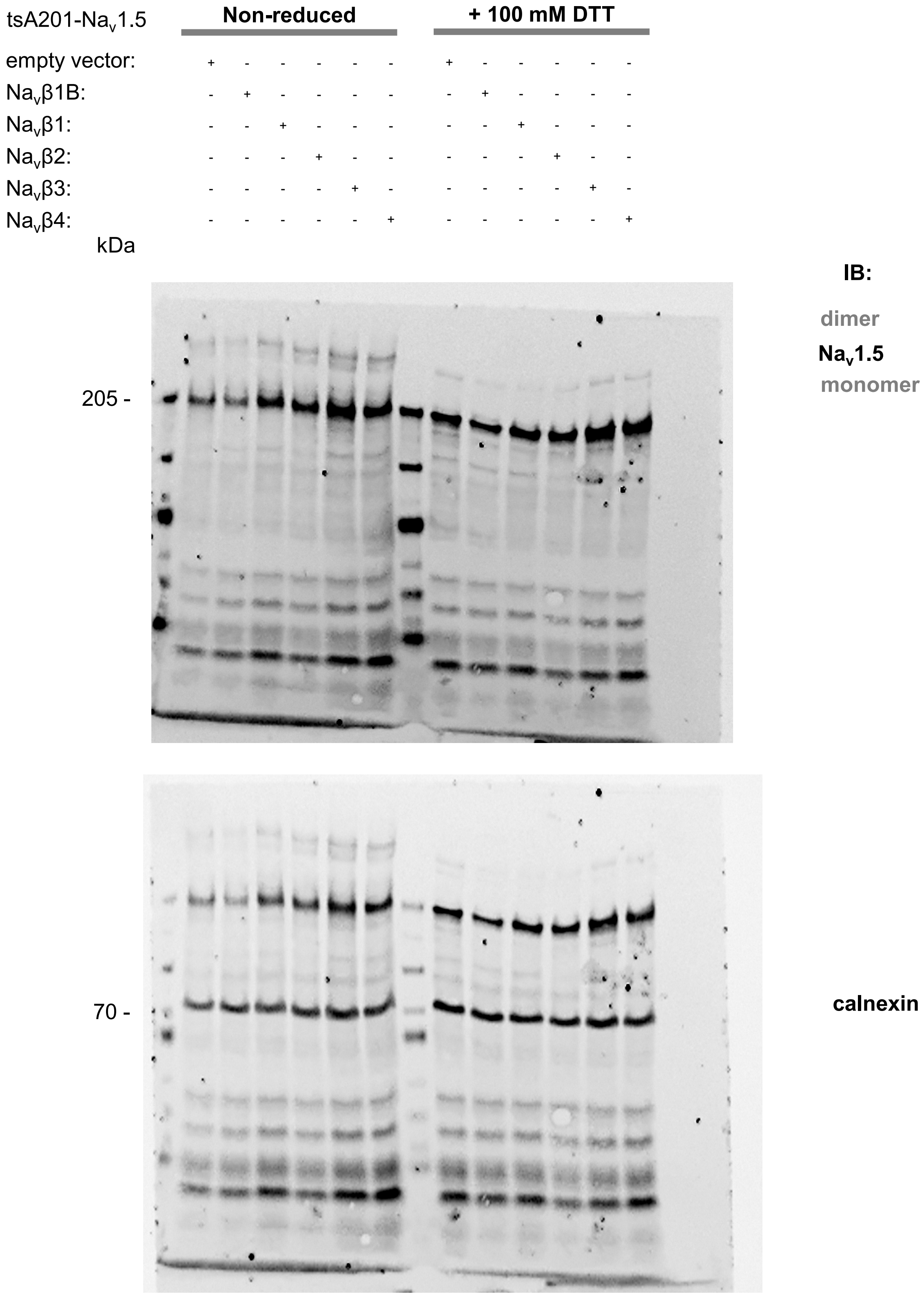

Fig3B (representative blot taken into analysis in Fig3D)

tsA201 WT

	INPUT (+100 mM DTT)							IP: FLAG (+100 mM DTT)						
3xFLAG-$Na_v1.5$:	+	+	+	+	+	+	-	+	+	+	+	+	+	-
3xHA-$Na_v1.5$:	+	+	+	+	+	+	+	+	+	+	+	+	+	+
$Na_v1.5$:	-	-	-	-	-	-	+	-	-	-	-	-	-	+
empty vector:	+	-	-	-	-	-	-	+	-	-	-	-	-	-
$Na_v\beta1B$:	-	+	-	-	-	-	-	-	+	-	-	-	-	-
$Na_v\beta1$:	-	-	+	-	-	-	+	-	-	+	-	-	-	+
$Na_v\beta2$:	-	-	-	+	-	-	-	-	-	-	+	-	-	-
$Na_v\beta3$:	-	-	-	-	+	-	-	-	-	-	-	+	-	-
$Na_v\beta4$:	-	-	-	-	-	+	-	-	-	-	-	-	+	-

IB:

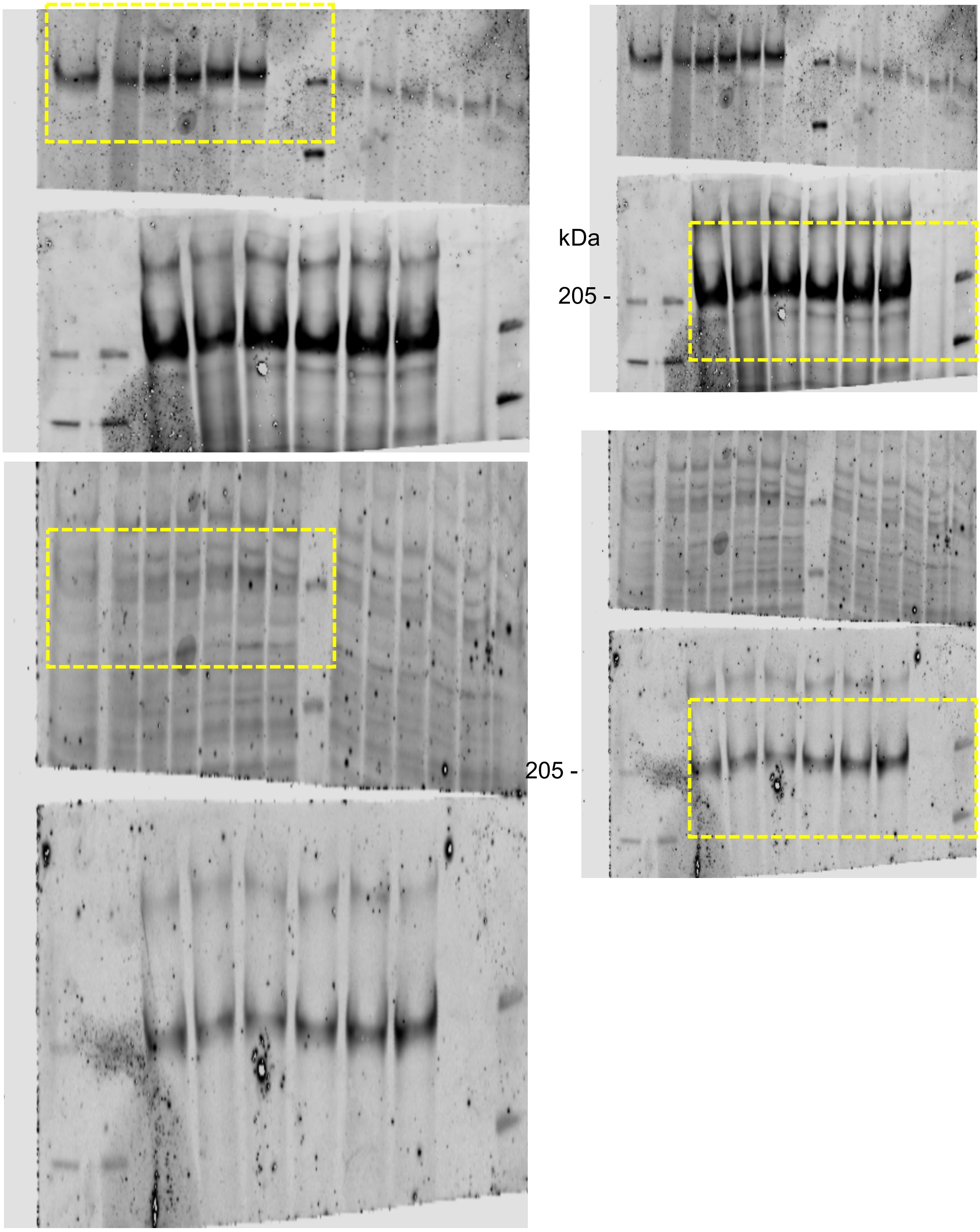

Fig3B (representative blot taken into analysis in Fig3D)

tsA201 WT

	INPUT (+100 mM DTT)							IP: FLAG (+100 mM DTT)						
3xFLAG-Na$_v$1.5:	+	+	+	+	+	+	-	+	+	+	+	+	+	-
3xHA-Na$_v$1.5:	+	+	+	+	+	+	+	+	+	+	+	+	+	+
Na$_v$1.5:	-	-	-	-	-	-	+	-	-	-	-	-	-	+
empty vector:	+	-	-	-	-	-	-	+	-	-	-	-	-	-
Na$_v$β1B:	-	+	-	-	-	-	-	-	+	-	-	-	-	-
Na$_v$β1:	-	-	+	-	-	-	+	-	-	+	-	-	-	+
Na$_v$β2:	-	-	-	+	-	-	-	-	-	-	+	-	-	-
Na$_v$β3:	-	-	-	-	+	-	-	-	-	-	-	+	-	-
Na$_v$β4:	-	-	-	-	-	+	-	-	-	-	-	-	+	-

IB:

kDa

50 -

30 -

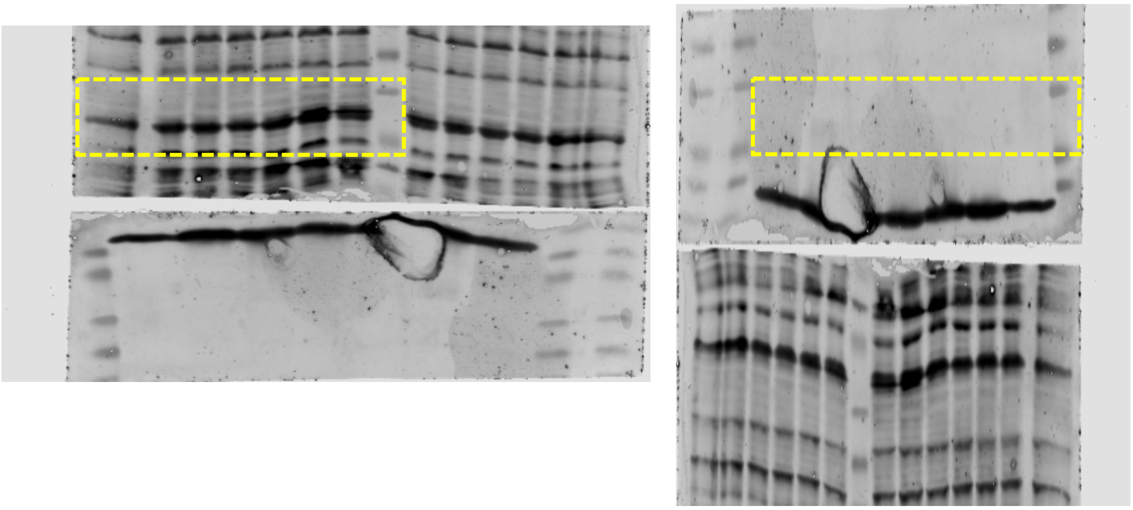

α-actin

Fig3B (additional repeat taken into analysis in Fig3D)

tsA201 WT	INPUT (+100 mM DTT)						IP: FLAG (+100 mM DTT)					
3xFLAG-$Na_v1.5$:	+	+	+	+	+	+	+	+	+	+	+	+
3xHA-$Na_v1.5$:	+	+	+	+	+	+	+	+	+	+	+	+
empty vector:	+	-	-	-	-	-	+	-	-	-	-	-
$Na_v\beta1B$:	-	+	-	-	-	-	-	+	-	-	-	-
$Na_v\beta1$:	-	-	+	-	-	-	-	-	+	-	-	-
$Na_v\beta2$:	-	-	-	+	-	-	-	-	-	+	-	-
$Na_v\beta3$:	-	-	-	-	+	-	-	-	-	-	+	-
$Na_v\beta4$:	-	-	-	-	-	+	-	-	-	-	-	+

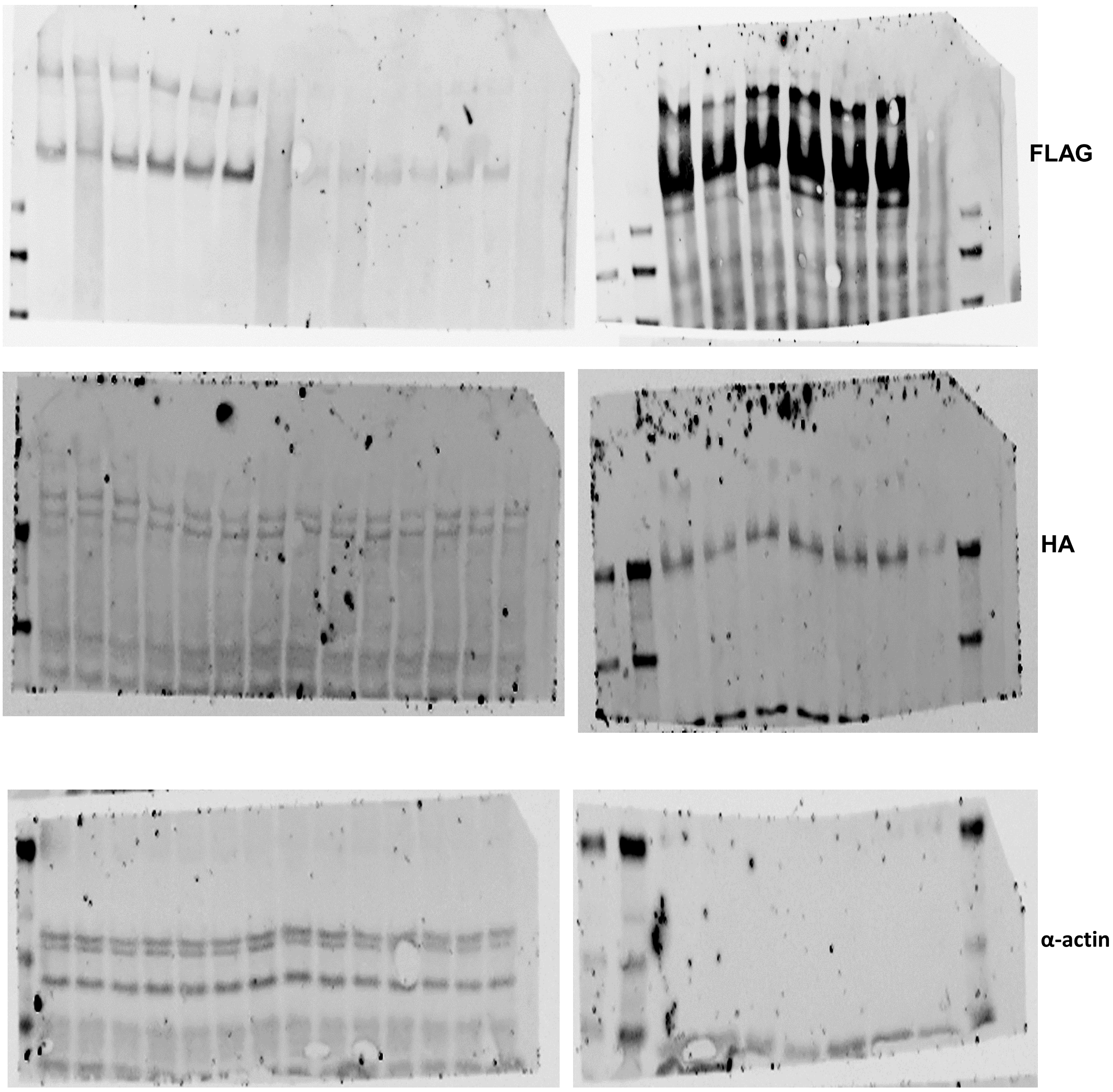

Fig3B (additional repeat taken into analysis in Fig3D)

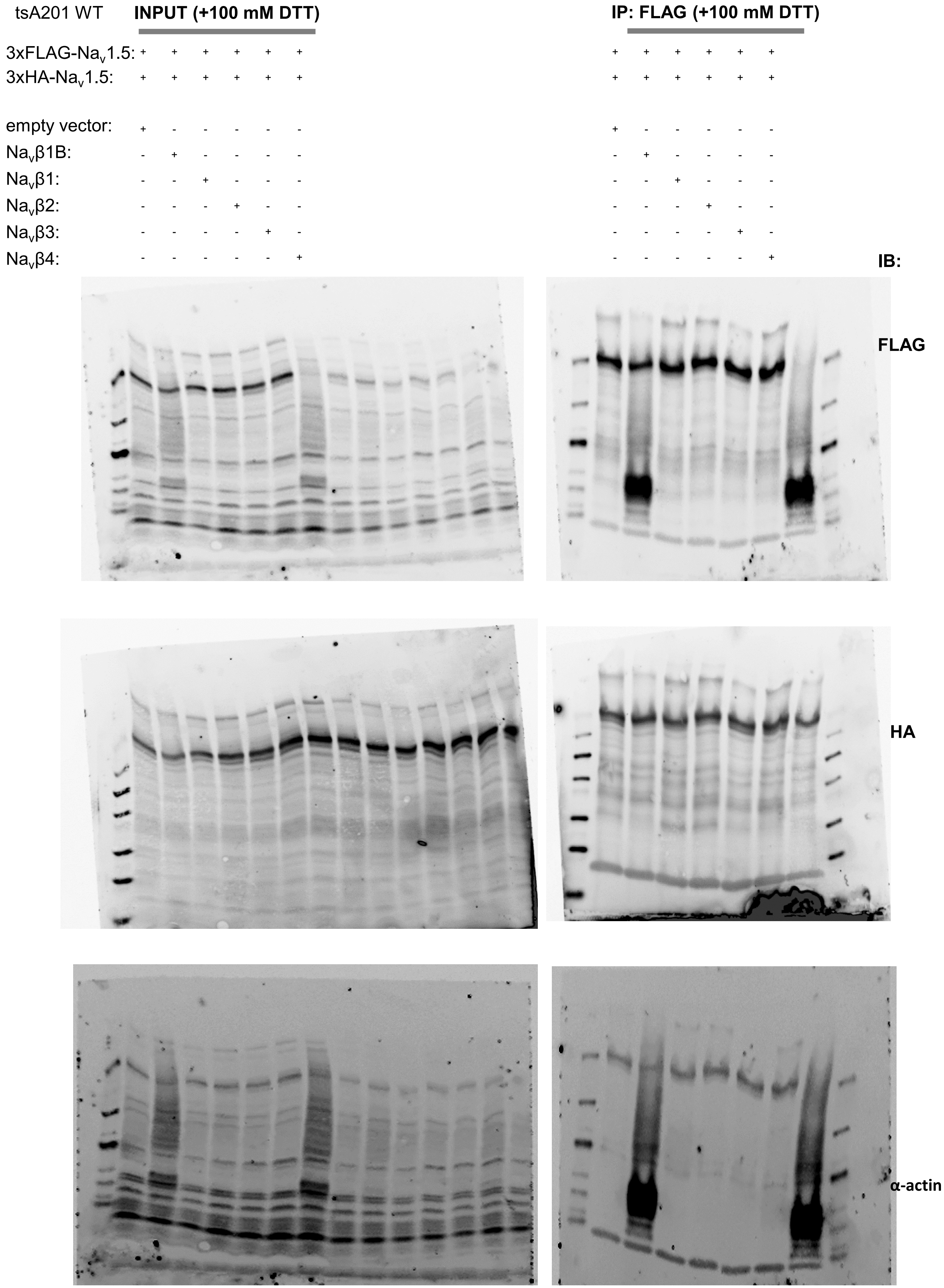

Supplement: Supplementary file 9 — Supplementary Material 9 [file 41598_2026_50463_MOESM9_ESM.pdf]
