# Supplementary material for: Biochemical assessment of α-α-subunit interactions of Nav1.5 in a heterologous expression system
Source: Sci Rep. 2026 May 4;16:20583. doi: 10.1038/s41598-026-50463-9 (PMC13333962; doi:10.1038/s41598-026-50463-9)

Fig4A representative blot taken into analysis in Fig4B)

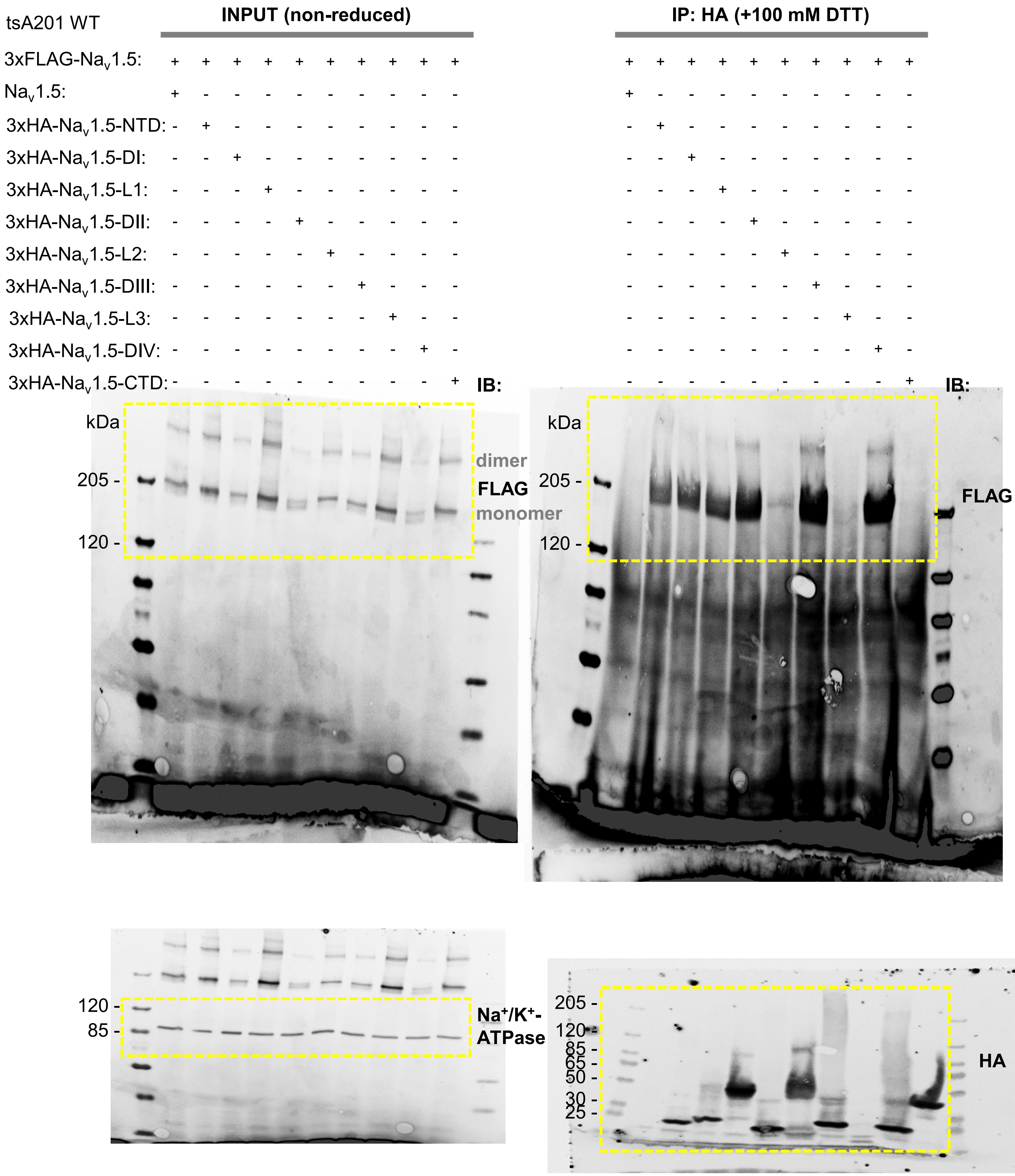

Fig4A (additional repeat taken into analysis in Fig4B)

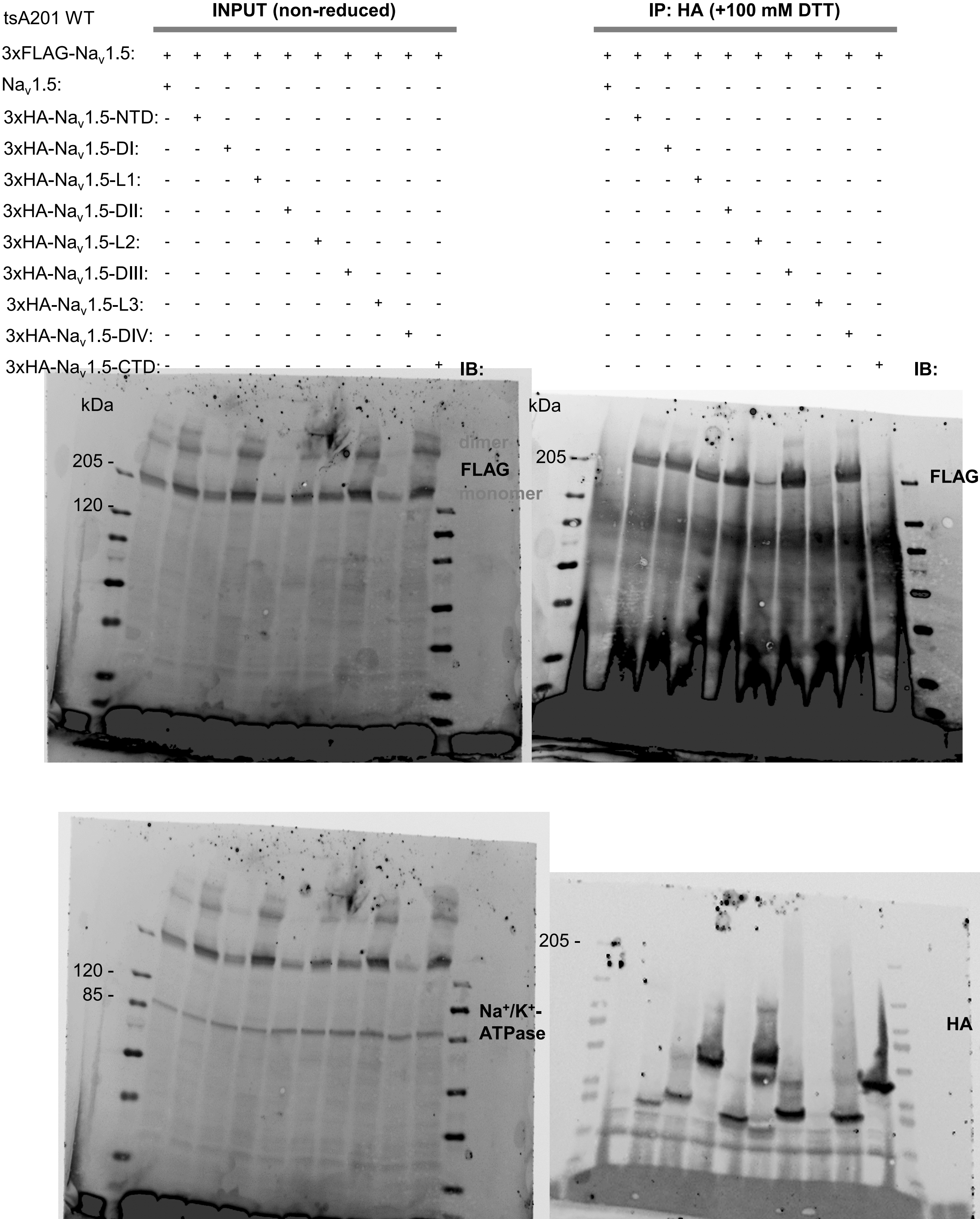

Fig4A (additional repeat taken into analysis in Fig4B)

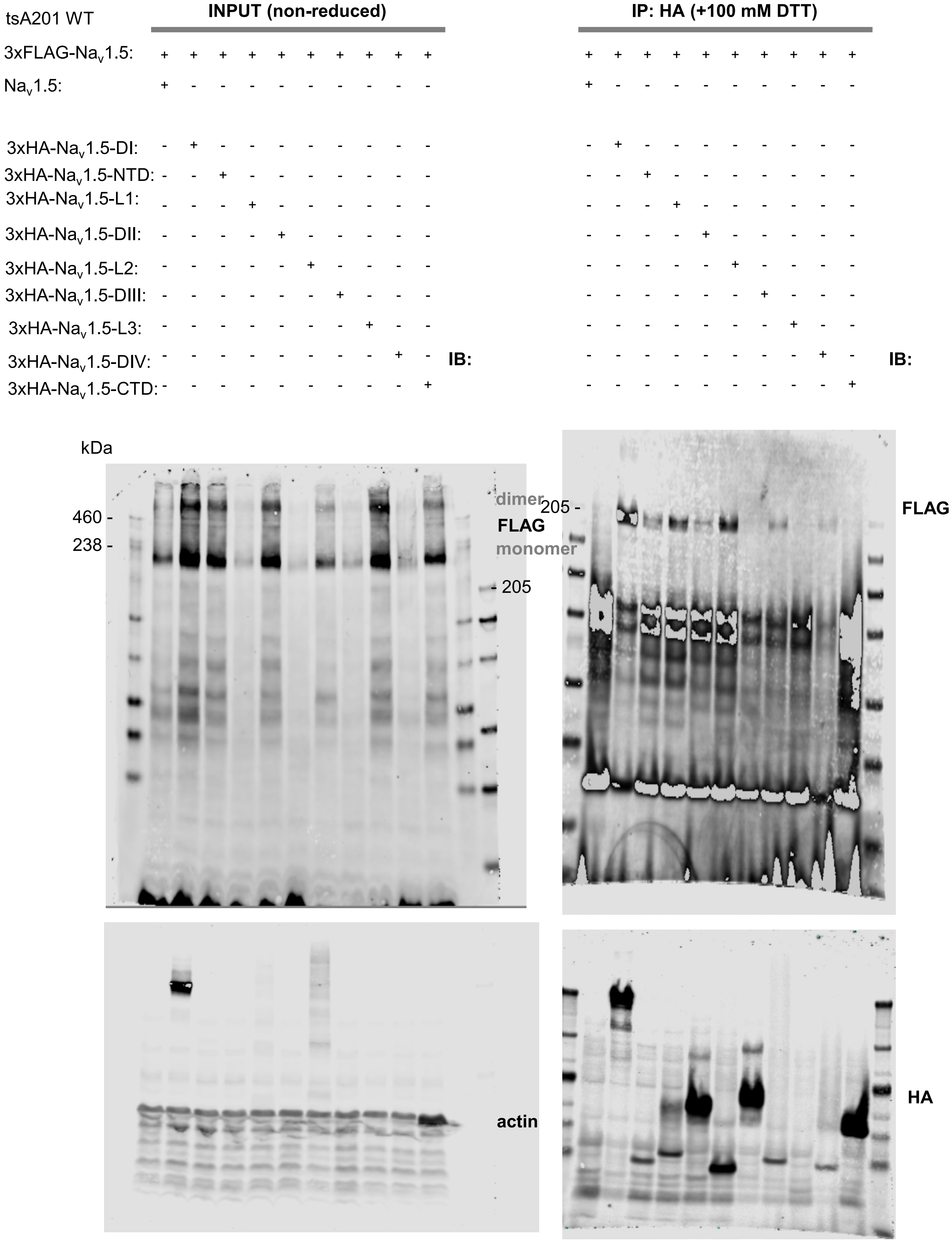

Supplement: Supplementary file 11 — Supplementary Material 11 [file 41598_2026_50463_MOESM11_ESM.pdf]
