# Supplementary material for: Biochemical assessment of α-α-subunit interactions of Nav1.5 in a heterologous expression system
Source: Sci Rep. 2026 May 4;16:20583. doi: 10.1038/s41598-026-50463-9 (PMC13333962; doi:10.1038/s41598-026-50463-9)

Fig5B upper panel (representative blot taken into analysis in Fig5C upper panel)

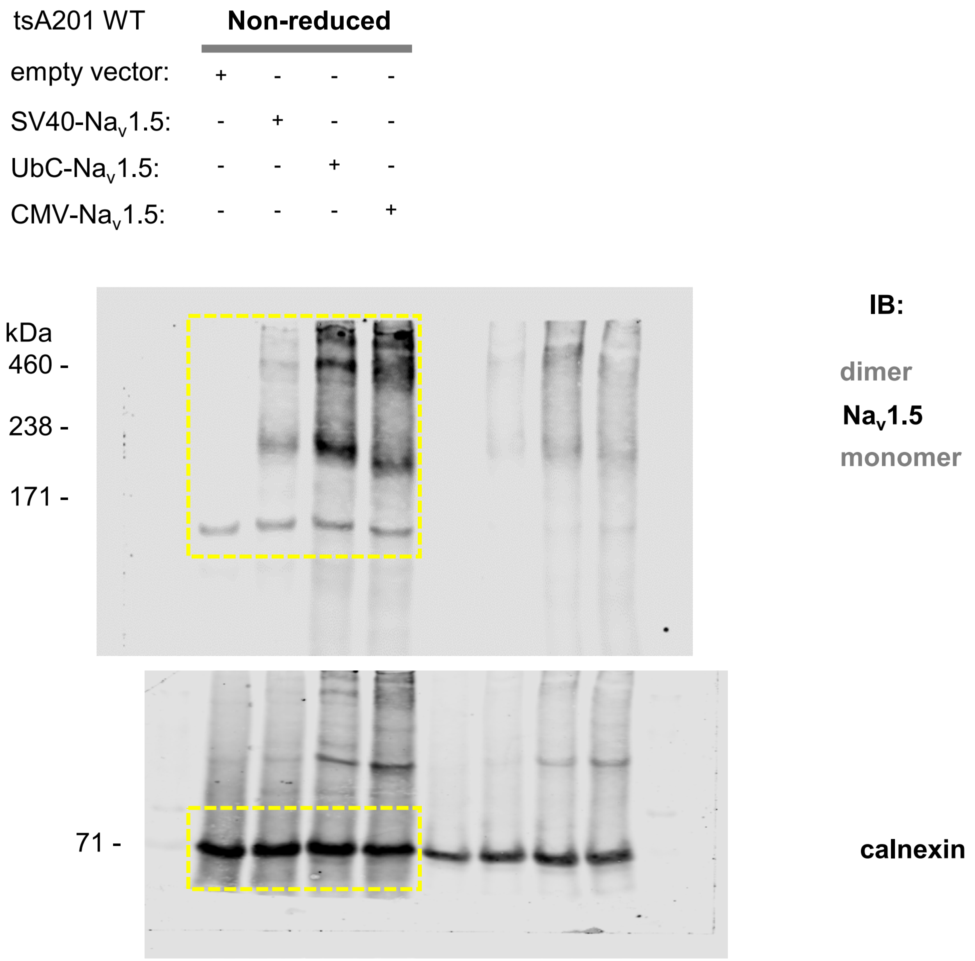

Fig5B upper panel (additional repeat taken into analysis in Fig5C upper panel)

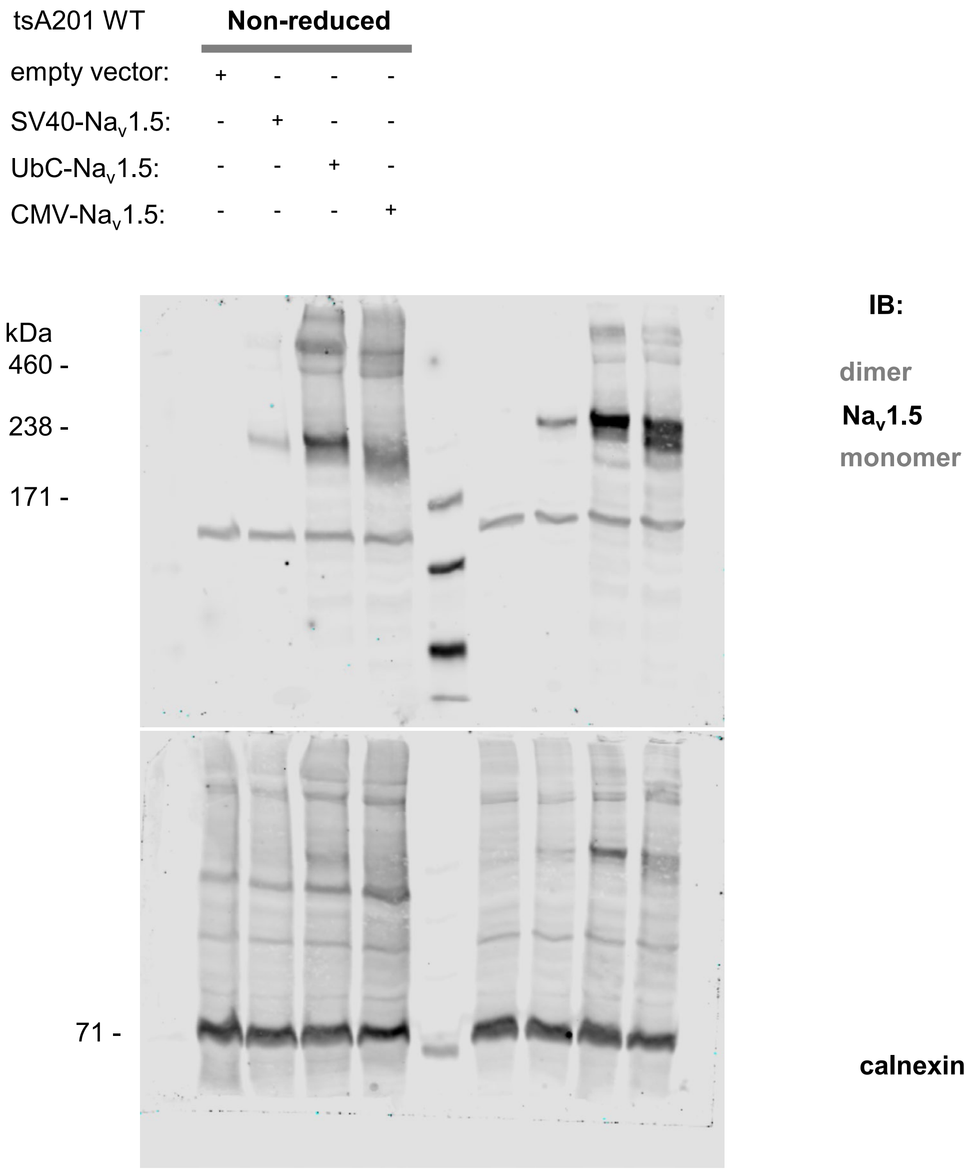

Fig5B upper panel (additional repeat taken into analysis in Fig5C upper panel)

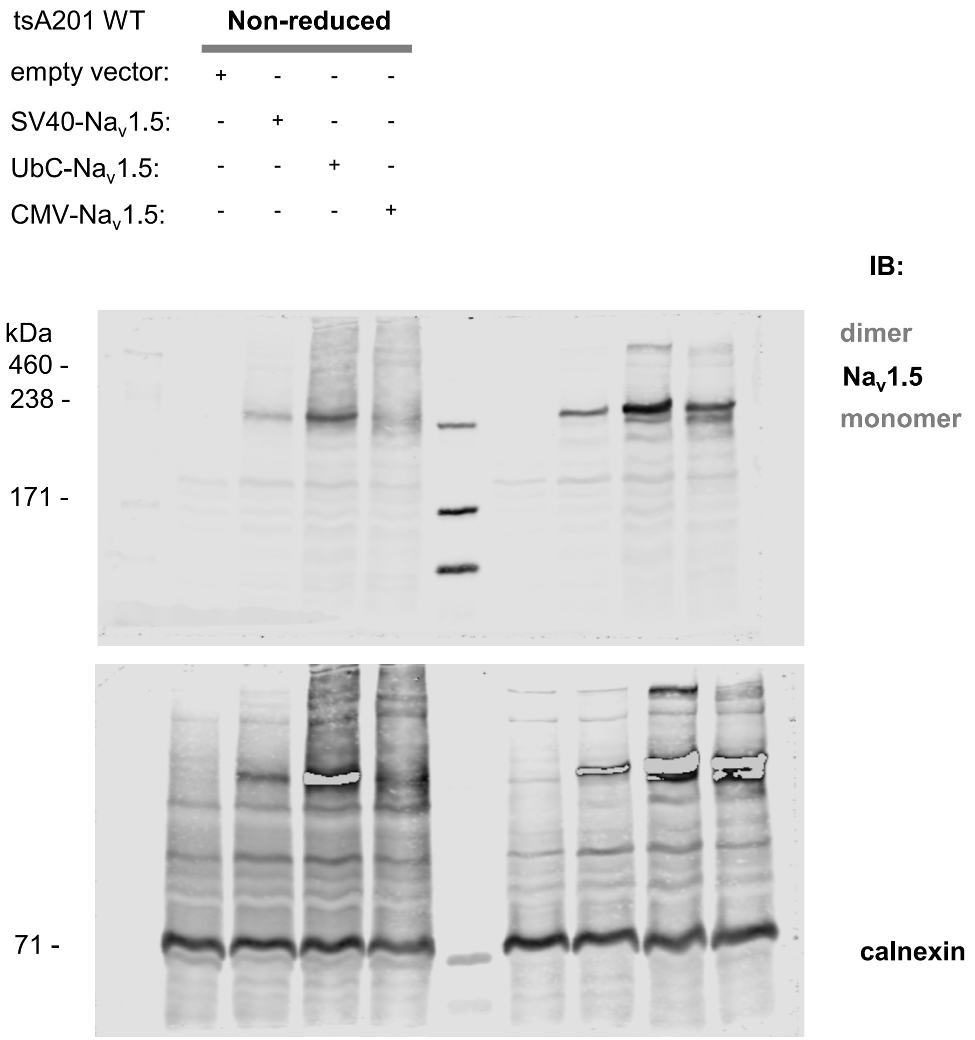

Fig5B upper panel (additional repeat taken into analysis in Fig5C upper panel)

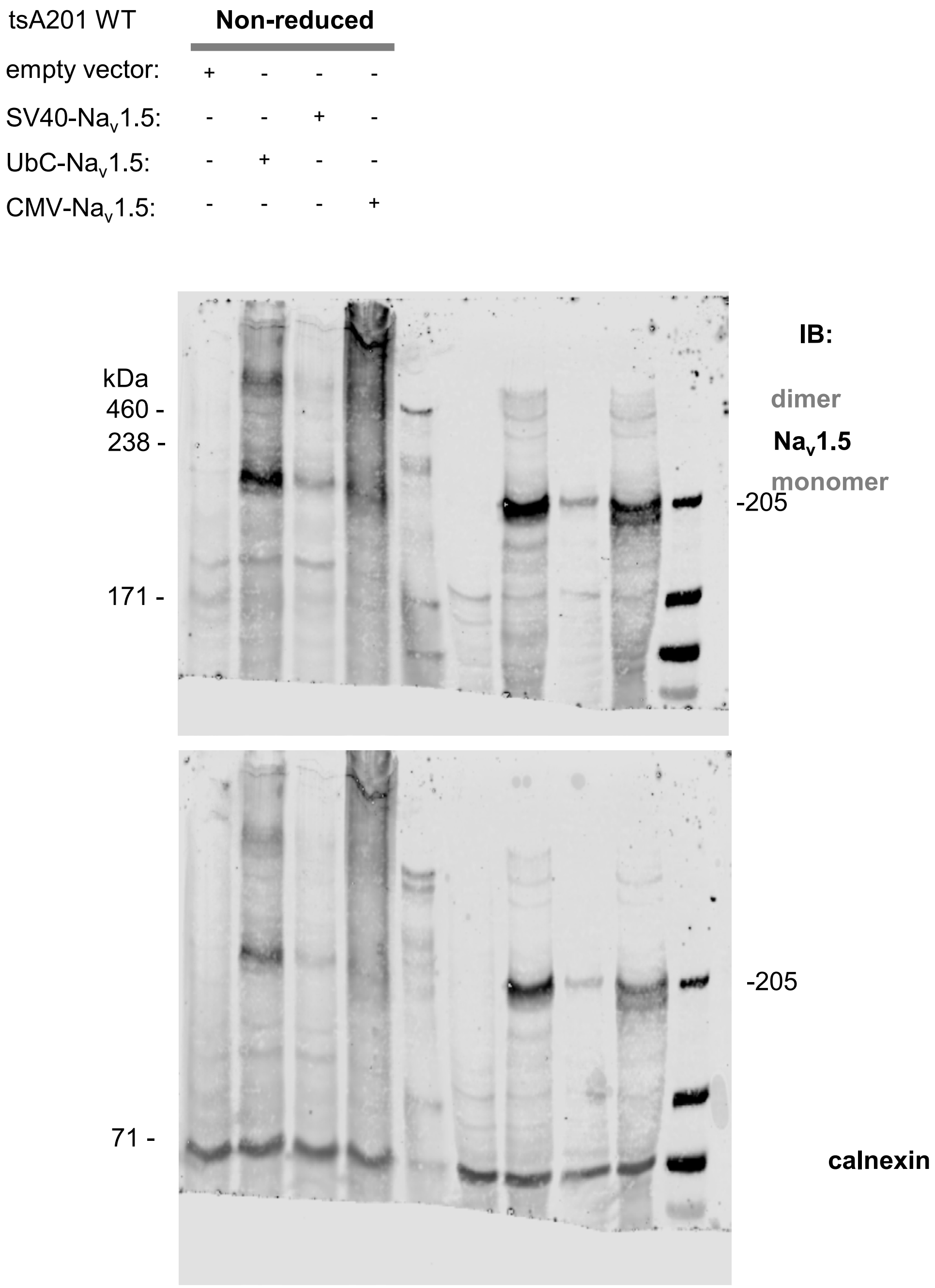

Fig5B bottom panel (representative blot taken into analysis in Fig5C bottom panel)

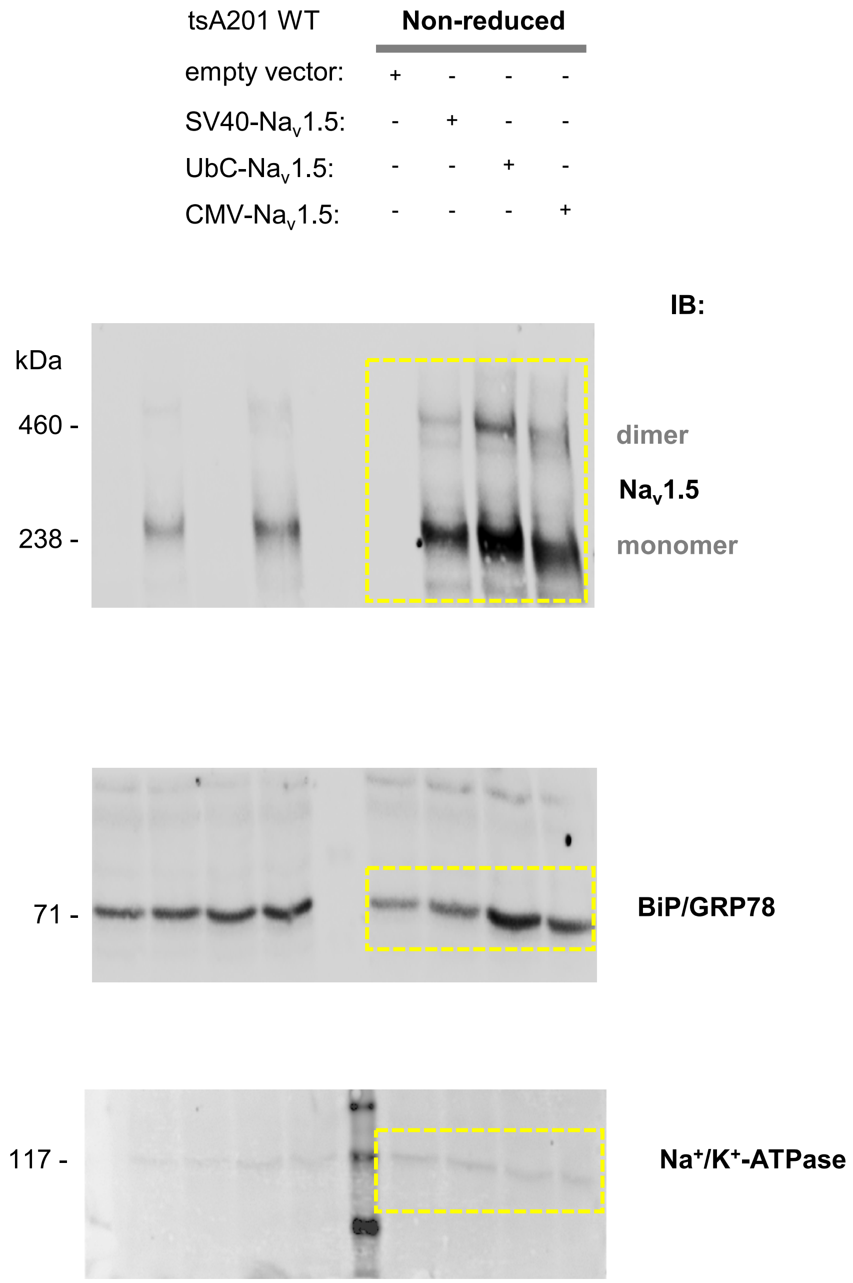

Fig5B bottom panel (additional repeat taken into analysis in Fig5C bottom panel)

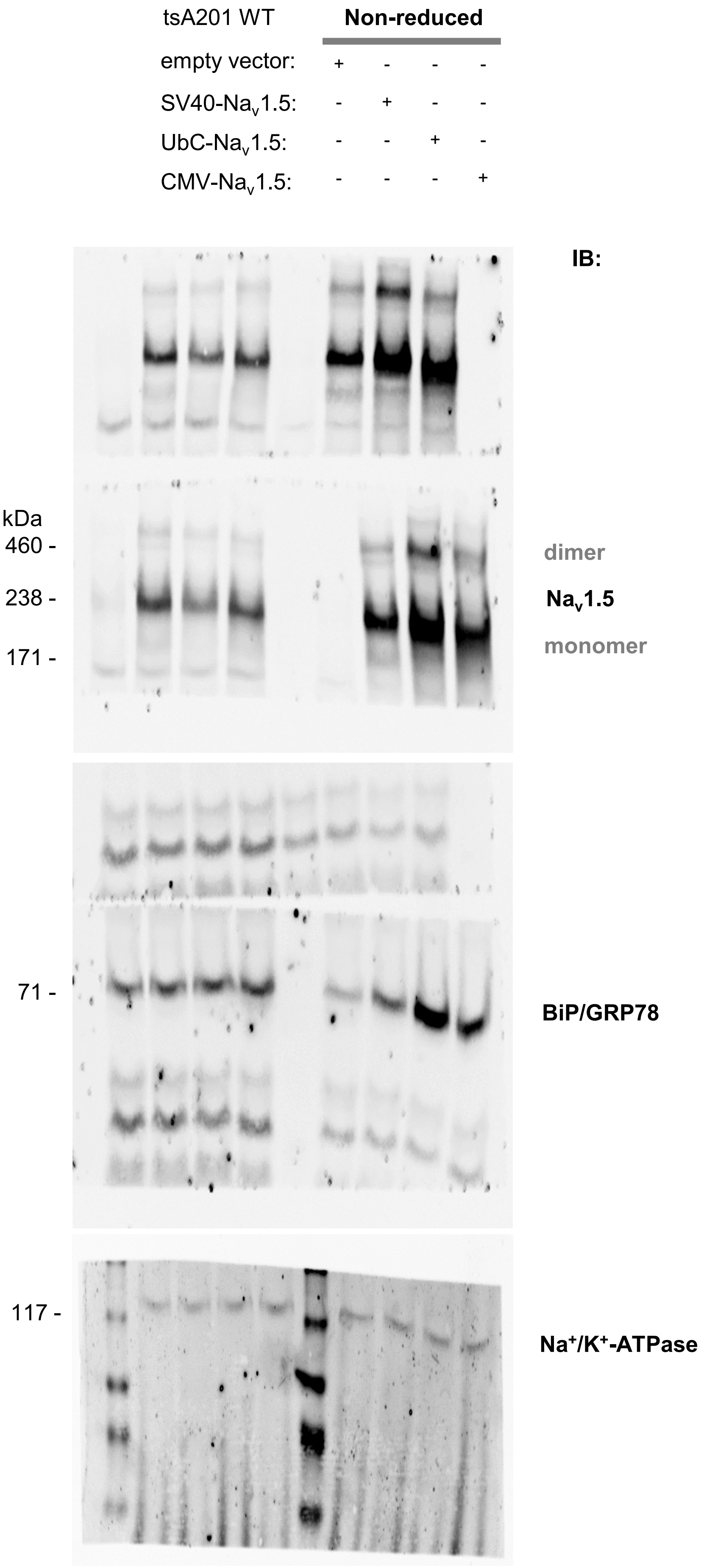

Fig5B bottom panel (additional repeat taken into analysis in Fig5C bottom panel)

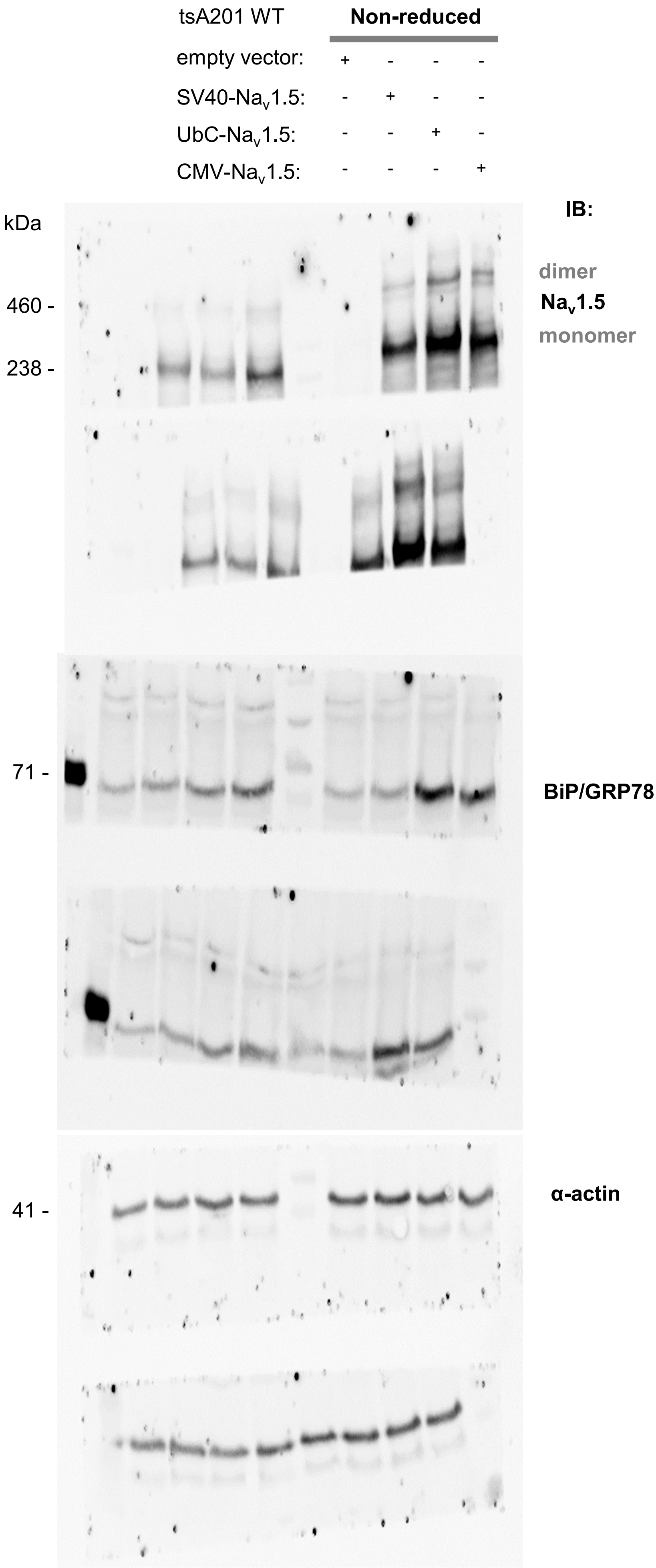

Fig5E (representative blot taken into analysis in Fig5F)

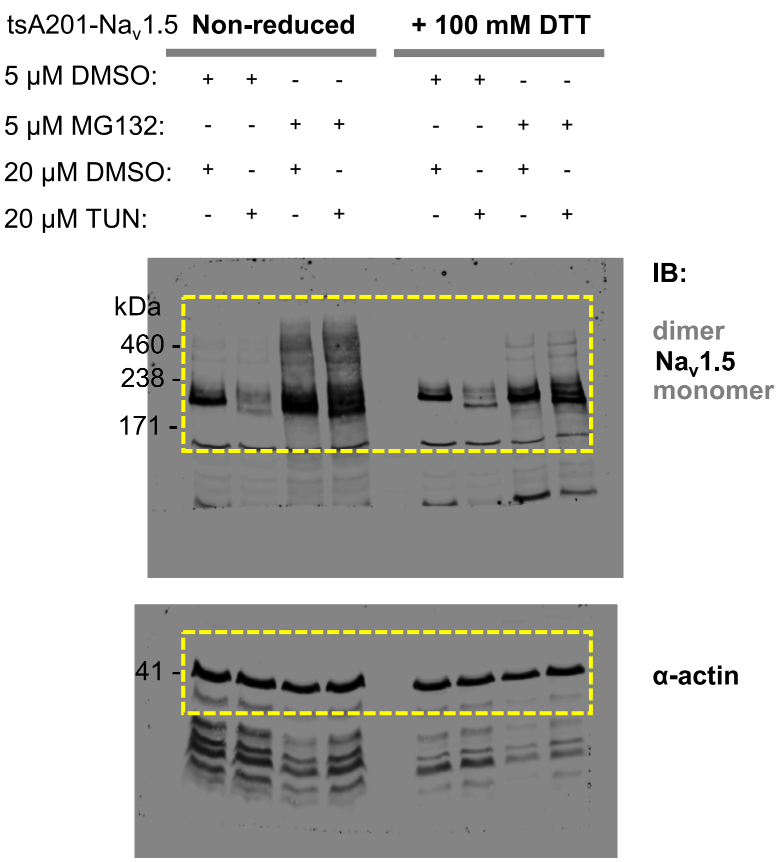

Fig5E (additional repeat taken into analysis in Fig5F)

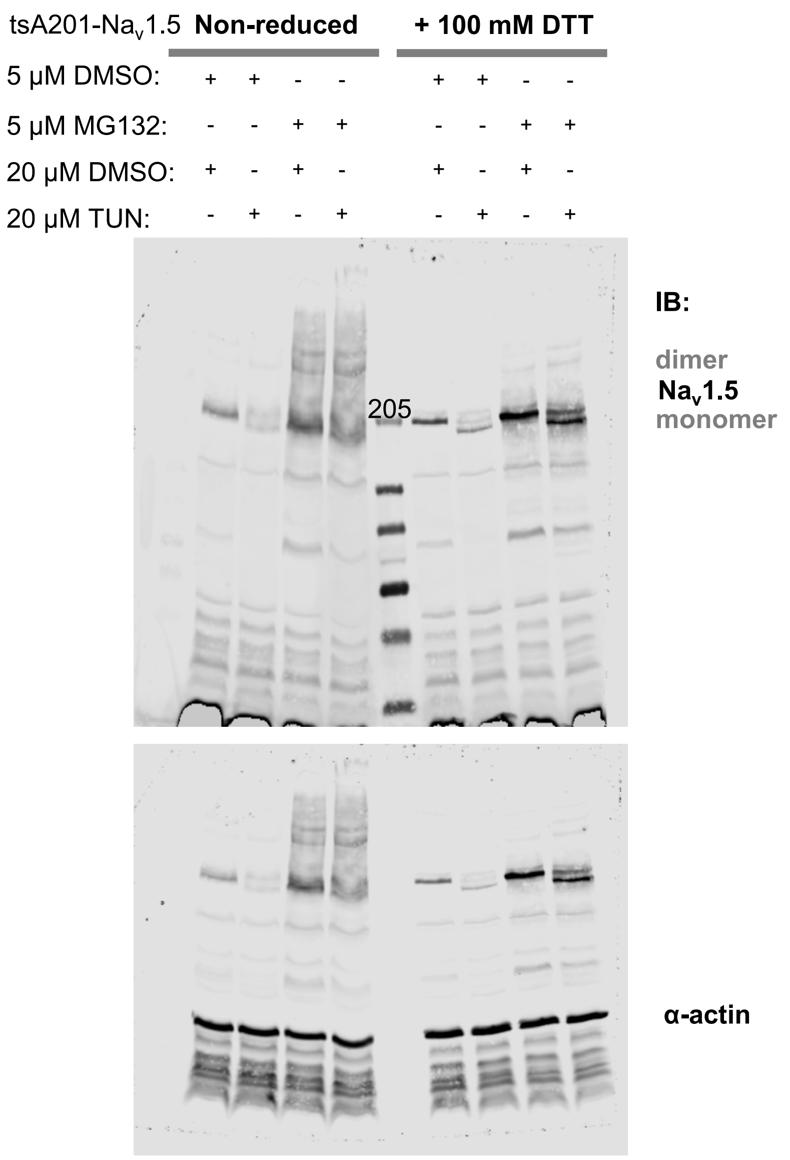

Fig5E (additional repeat taken into analysis in Fig5F)

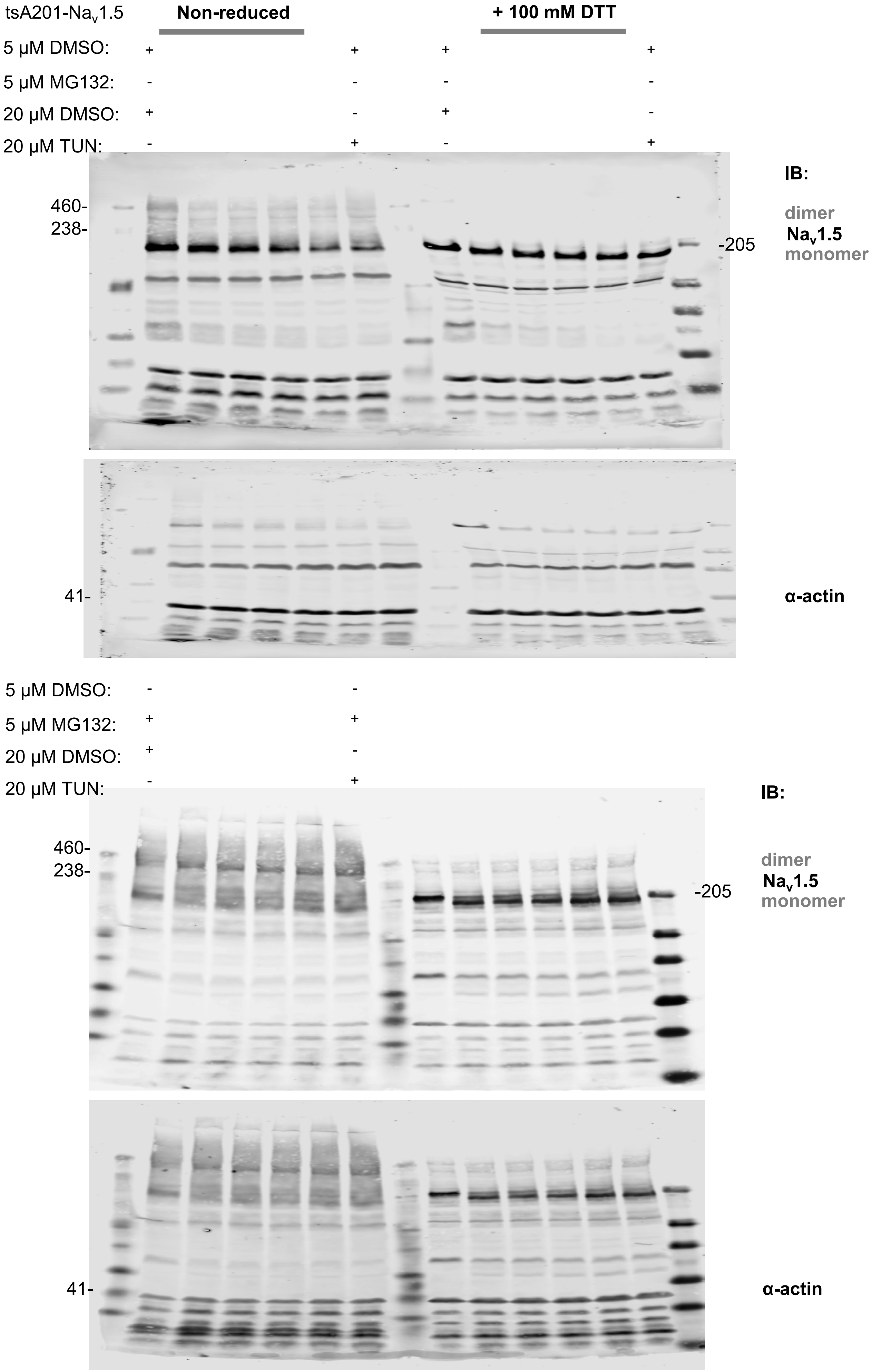

Supplement: Supplementary file 15 — Supplementary Material 15 [file 41598_2026_50463_MOESM15_ESM.pdf]
